# Krt14 and Krt15 differentially regulate regenerative properties and differentiation potential of airway basal cells

**DOI:** 10.1172/jci.insight.162041

**Published:** 2023-01-24

**Authors:** Vitaly Ievlev, Thomas J. Lynch, Kyle W. Freischlag, Caitlyn B. Gries, Anit Shah, Albert C. Pai, Bethany A. Ahlers, Soo Park, John F. Engelhardt, Kalpaj R. Parekh

**Affiliations:** 1 Department of Anatomy & Cell Biology, University of Iowa, Carver College of Medicine, Iowa City, Iowa, USA.; 2 Department of Cardiothoracic Surgery, University of Iowa Hospitals and Clinics, Carver College of Medicine, Iowa City, Iowa, USA.

**Keywords:** Pulmonology, Stem cells, Adult stem cells, Mouse models, Respiration

## Abstract

Keratin expression dynamically changes in airway basal cells (BCs) after acute and chronic injury, yet the functional consequences of these changes on BC behavior remain unknown. In bronchiolitis obliterans (BO) after lung transplantation, BC clonogenicity declines, which is associated with a switch from keratin15 (Krt15) to keratin14 (Krt14). We investigated these keratins’ roles using Crispr-KO in vitro and in vivo and found that Krt14-KO and Krt15-KO produce contrasting phenotypes in terms of differentiation and clonogenicity. Primary mouse Krt14-KO BCs did not differentiate into club and ciliated cells but had enhanced clonogenicity. By contrast, Krt15-KO did not alter BC differentiation but impaired clonogenicity in vitro and reduced the number of label-retaining BCs in vivo after injury. Krt14, but not Krt15, bound the tumor suppressor stratifin (Sfn). Disruption of Krt14, but not of Krt15, reduced Sfn protein abundance and increased expression of the oncogene dNp63a during BC differentiation, whereas dNp63a levels were reduced in Krt15-KO BCs. Overall, the phenotype of Krt15-KO BCs contrasts with Krt14-KO phenotype and resembles the phenotype in BO with decreased clonogenicity, increased Krt14, and decreased dNp63a expression. This work demonstrates that Krt14 and Krt15 functionally regulate BC behavior, which is relevant in chronic disease states like BO.

## Graphical abstract

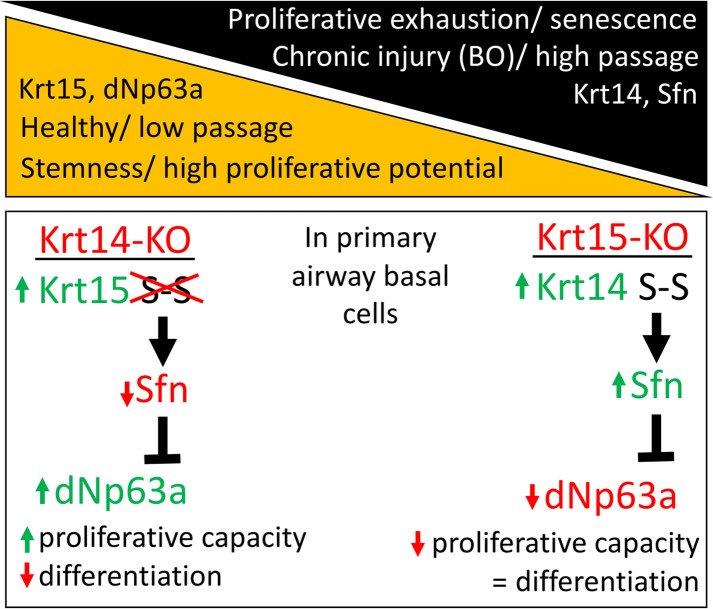

## Introduction

Respiratory diseases are among the most prevalent causes of death in the United States (1). Lung transplantation is the only effective treatment option for many end-stage lung diseases; however, greater than 80% of lung transplant recipients develop chronic lung allograft dysfunction (CLAD) within 12 years of the operation (2). The most common form of CLAD is bronchiolitis obliterans (BO), which manifests in fibrotic occlusion of small airways (3). Airway epithelial injury is a risk factor for BO (3), which suggests that a failure in airway regeneration by stem cells likely contributes to the BO disease pathoprogression. It has been demonstrated that depletion of airway epithelial basal stem cells precedes formation of BO-like lesions in murine tracheas after Cl_2_ gas exposure (4). Airway basal stem cells can be depleted as a result of their exhaustion over persistent cycles of epithelial repair, because of chronic graft injury caused by infections, inflammation, and immune responses of the host (5).

Airway basal cells (BCs) are recognized as resident stem cells of the surface airway epithelium (SAE) that can migrate, divide, and differentiate after an injury (6). They are defined by expression of keratin5 (Krt5), a type II keratin that must heterodimerize with a type I keratin to form intermediate filaments (IFs). Krt5 is the predominant type II keratin expressed in BCs and can pair with either Krt14 and Krt15 (7). Krt15 expression in BCs of uninjured airways has been suggested by single-cell RNA-Seq, whereas Krt14 expression is infrequent on the surface of homeostatic airways but is abundant in submucosal glands (SMGs), a major secretory compartment and a niche for reserve airway stem cells (8, 9).

Our group and others observed that Krt14^+^ cell abundance increases on the airway surface in multiple lung pathologies, such as in idiopathic pulmonary fibrosis, BO occurring after lung transplantation, restrictive allograft syndrome (RAS) after lung transplantation, and in injury models like SO_2_ and Cl_2_ gas exposure, and injuries caused by naphthalene and polidocanol exposure (8, 10–14). It is unclear, however, whether Krt14 appearance in the SAE reflects migration and expansion of glandular progenitors that express Krt14 at baseline or a change within surface airway BCs to upregulate Krt14 expression. During a chronic injury, such as in BO, Krt14 appears in the surface airway BCs, which correlates with a decline in their proliferative capacity in vitro (14). However, it is unknown whether this change in keratin expression alone can affect basal stem cell properties and fate. In this article, we present a paradigm to contextualize Krt14 expression dynamics and also evaluate Krt15 as the basal keratin alternative to Krt14. We show that Krt14 and Krt15 expression dynamics have a functional impact on BC behavior (namely, proliferation, maintenance, and differentiation).

Overall, Krt14 and Krt15 are similar in their amino acid composition (approximately 81% amino acid homology); however, there are important biochemical differences between them. For example, Krt14 has a cysteine residue in position 373 (alanine in Krt15), which has been shown to form interfilamentous disulfate bonds (15–17). These cross-linkages alter nuclear shape and act as recruitment sites for signal cascades in differentiating keratinocytes (15). This mechanism might be conserved in airway BCs as well.

We hypothesized that changes in Krt14 and Krt15 expression not only indicate the regenerative state of airway BCs but also actively influence cell fate decisions. In this work, we demonstrate that disruption of Krt14 and Krt15 in mouse airway BCs leads to opposite phenotypes. Specifically, the loss of Krt14 enhances the cells’ proliferative capacity but impairs ciliated and club cell differentiation, whereas the loss of Krt15 impairs proliferative capacity and does not impede differentiation. This change is accompanied by nuclear enlargement and elongation in differentiating Krt14-KO, but not in Krt15-KO BCs. These differentiating Krt14-KO BCs have decreased levels of the tumor suppressor stratifin (Sfn; also known as 14-3-3σ) and upregulated levels of the oncogene dNp63a. By contrast, Krt15-KO primary airway BCs have a decreased dNp63a expression early in their differentiation. Krt14 whole-body KO have poor neonatal survival, reduced BW, visually smaller lungs and tracheal SMGs, as well as decreased club cell abundance. Krt15-KO mice, conversely, appear phenotypically normal with the exception of a mild hair loss defect. We additionally observed a decrease in label-retaining tracheal BCs 21 days after injury in Krt15-KO mice. Last, we show that the phenotype observed in Krt15-KO BCs functionally resembles the phenotype of BO after lung transplantation (14), in which BCs have increased Krt14, decreased Krt15 and dNp63a expression, and impaired proliferative capacity. Cumulatively, these data suggest that Krt14 and Krt15 influence BC behavior that controls proliferation, quiescence, and/or differentiation in response to injury.

## Results

### Krt14 and Krt15 expression dynamically changes after airway injury in vivo and in vitro.

Upregulation of Krt14 expression in the SAE has been reported in multiple lung pathologies and injury models (8, 10–14). However, considering that keratins are obligate heterodimers and Krt5 (type II) is expressed at homeostasis, it remains unclear which other type I keratin serves as a heterodimerization partner to Krt5 at homeostasis in the absence of Krt14. To begin answering this question, we analyzed the published single-cell RNA-Seq literature and identified Krt15 as a likely candidate (18–22). Additionally, Krt15 is expressed in murine tracheal BCs (23). To verify these previous findings and extend them to humans and ferrets, we successfully localized Krt15 and Krt14 in the large airways of mice, ferrets, and humans. At homeostasis, Krt15 expression was mostly limited to BCs on the airway surface and in the gland ducts; Krt14 was expressed in gland ducts and in the myoepithelial cells (MECs) of gland tubules and acini (Supplemental Figure 1, A–E; supplemental material available online with this article; https://doi.org/10.1172/jci.insight.162041DS1). α-SMA was absent on the airway surface and in the gland ducts but present in the MECs surrounding tubules and acini at homeostasis, a finding consistent among mice, ferrets, and humans (Supplemental Figure 1, A–D).

**Figure 1:**
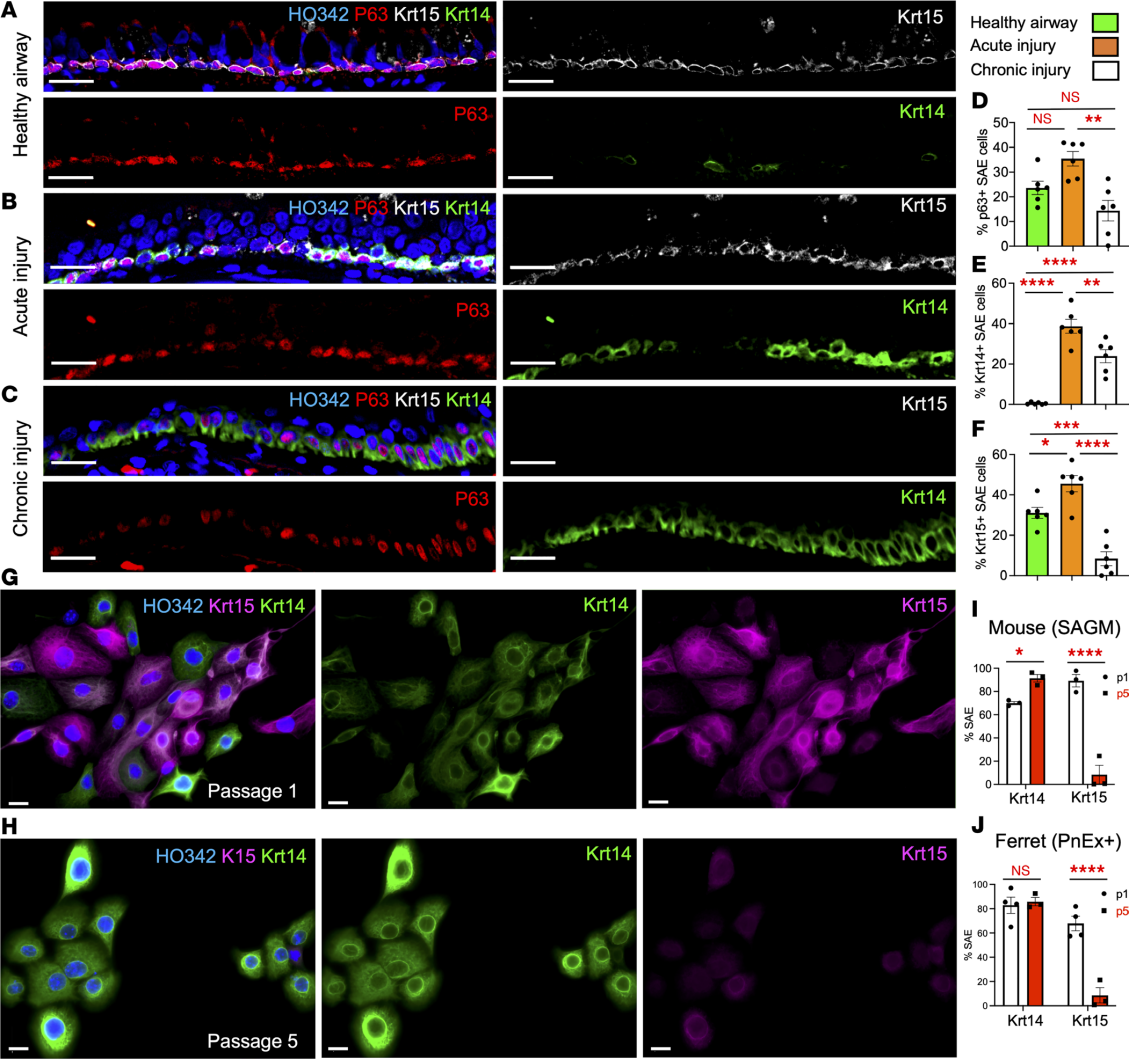
Dynamic changes in Krt14 and Krt15 expression after an injury and in vitro. (**A**–**C**) Immunofluorescence staining of a cartilaginous ferret airway from a healthy control ferret (**A**), a ferret 3 days after polidocanol injury (**B**), and a ferret that developed a form of CLAD after lung transplantation (**C**). (**D**–**F**) Quantified protein expression of p63 (**D**), Krt14 (**E**), and Krt15 (**F**) in large airways. (**G** and **H**) Murine SAE cells show a different basal keratin profile on passage 1 (**G**) compared with passage 5 (**H**). (**I** and **J**) Quantification of Krt14 and Krt15 abundance in primary SAE from mice (**I**) and ferrets (**J**) grown with dual SMAD inhibition in SAGM and Pneumacult-Ex Plus (PnEx+), respectively. Graphs show mean data ± SEM; *n* = 6 ferrets (**D**–**F**) or *n* ≥ 3 cell pools (**I** and **J**). Significance was determined by 1-way ANOVA and Tukey multiple comparison test (**D**–**F**) or by 2-way ANOVA and Sidak multiple comparison test (**I** and **J**). *P < 0.05, **P < 0.01, ***P < 0.001, ****P < 0.0001. Scale bars: 50 μm in **A**–**C** and 20 μm in **G** and **H**.

Next, we confirmed that Krt14 and Krt15 dynamically change their expression in several injury model systems. We observed that at homeostasis, Krt15 is a predominant type I keratin in the ferret airway surface BCs, whereas Krt14 expression on the airway surface is infrequent (Figure 1A and Supplemental Figure 1). Krt14 dramatically expands its expression domain as early as 3 days after a polidocanol injury in ferrets, with continued Krt15 expression (Figure 1B). In chronic and persistent lung injuries, such as in RAS and BO, Krt15 expression largely disappears, whereas Krt14 persists in BCs of large airways (Figure 1, C–F). We observed a similar trend in human OB after lung transplantation, where Krt14 expressing BCs become more abundant and Krt15-expressing BCs decline in chronically injured airways of patients with BO compared with control patients with no CLAD (Supplemental Figure 2, A and B). The regions contrasting Krt14 and Krt15 expression showed an apparent decrease in Krt15 staining intensity and stratification of Krt14^+^ regions in human BO (Supplemental Figure 2, C, D, F, and G). Morphometric quantification confirmed that OB airway surfaces contained fewer Krt15^+^ and more Krt14-expressing cells than no-CLAD airways (Supplemental Figure 2E). BO diagnosis in patients was made on the basis of computed tomography scans, pulmonary function tests, and H&E histology of the lung specimens (Supplemental Figure 3).

For BC functional studies, it was important to establish a baseline for Krt14 and Krt15 expression in vitro. Cell culture conditions are often compared with a constant wound healing state; therefore, we were not surprised to observe that Krt14 is expressed in the majority of primary airway BCs as early as 7 days in culture (passage 1) (Figure 1, G and I) and becomes ubiquitously expressed by passage 5 (Figure 1, H and I). By contrast, Krt15 is expressed in almost all of the primary BCs on passage 1 (Figure 1, E and I) and is gradually lost in the majority of BCs by passage 5 (Figure 1, H and I). These trends were similar between mouse and ferret SAE, which was cultured in Small Airway Growth Medium (SAGM) and Pneumacult-Ex Plus medium, respectively (Figure 1, I and J). Overall, these changes resembled the trends observed during injury regeneration in vivo.

### Krt14 and Krt15 expression during wound healing in an ex vivo tracheal injury model.

**Figure 2:**
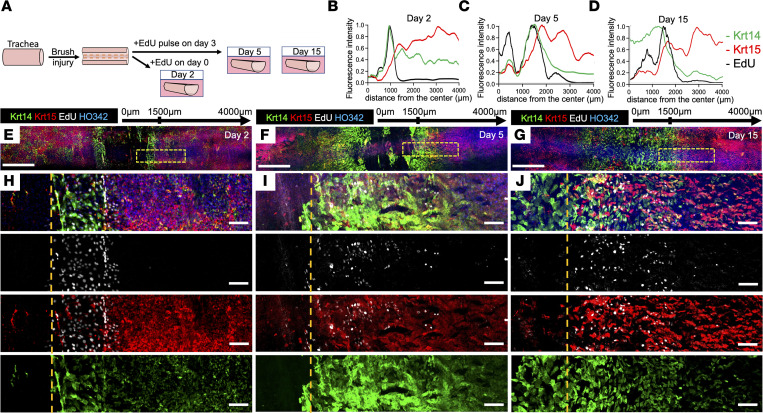
Krt14 is upregulated at the edges of a regenerating wound ex vivo. (**A**) Experimental design: ferret tracheal explants were excised, cut longitudinally along the membranous portion of the trachea, and cultured submerged in F-medium for the indicated time. Day 2 samples were cultured with EdU starting on day 0. Remaining samples were pulsed with EdU for 24 hours on day 3. (**B**–**D**) Staining intensity plots of the right halves of the explants collected on days 2, 5, and 15 after injury. The *x*-axis represents the distance from the center of the scratch. (**E**–**G**) Overview micrographs of the scratch on days 2, 5, and 15 after injury. (**H**–**J**) Close-up micrographs of the boxed regions from the explants on day 2 (**H**), day 5 (**I**), and day 15 (**J**). Images are representative of ≥3 representative experiments with explants from different animals. Orange dashed lines represent the original wound edge. Scale bars: 500 μm in **E**–**G** and 50 μm in **H**–**J**.

The question remained whether the increased abundance of Krt14^+^ BCs in the SAE during wound healing was due to the migration of Krt14^+^ progenitors from glands or enhanced Krt14 expression of resident BCs in the SAE. To address this question, we used an ex vivo brush-injury model of the ferret trachea to examine dynamic changes in keratin expression in a highly controlled setting (Figure 2A). We hypothesized that migration of Krt14^+^ gland progenitors onto the surface and upregulation of Krt14 in resident SAE BCs were both responsible for the increased abundance of Krt14+ BCs. We observed that on day 2 after partial brushing of the luminal surface of the explant, Krt14 staining was more intense at the edges of the scratch (see the area to the left of dotted orange line in Figure 2, H–J) in cells that incorporated ethynyl-deoxy-uridine (EdU) and also expressed lower levels of Krt15 (Figure 2, B, E, and H). By day 5 after injury, the expression domain of Krt14 widened and correlated with a broader band of EdU incorporation (Figure 2, C, F, and I). By day 15, we noticed that the center of the scratch, which was already covered with Krt14^+^ cells, retained less EdU (pulsed for 24 hours on day 3) than the periphery of the scratch marked by higher levels of Krt15 expression (Figure 2, D, G, and J). This suggests that most of the Krt14^+^ cells in the center of the scratch either proliferated after the EdU pulse or had diluted the EdU to the point that it became undetectable. By contrast, Krt15^+^ cells that stayed approximately at the initial scratch boundary retained high levels of EdU. This observation suggests that Krt14/Krt15 expression might distinguish the transit amplifying progenitor fate of BCs from the label-retaining stem cell fate.

To test whether the increased Krt14 abundance on the airway surface is partly due to migration of Krt14^+^ gland progenitors, we removed the entire SAE by brushing. We observed Krt14^+^ epithelial cells emerge onto the airway surface from SMGs during the 15-day recovery, which was enough to re-epithelialize the majority of the explant surface (Supplemental Figure 4, A–D). The re-epithelialized airway surface on day 15 after injury (DPI) 15 had overall larger cells, which were mostly Krt14^+^/Krt15^–^, unlike in the uninjured explants on days 2 and 15, which had compact Krt15^+^ cells (Supplemental Figure 4, E–G). We additionally observed Krt14^+^/α-SMA^+^ MECs on the surface of injured explants on day 15 (Supplemental Figure 4H). Thus, we conclude that glandular contribution to the airway surface repair can at least partially account for appearance of Krt14 on the injured surface epithelium. These observations are consistent with prior work demonstrating surface repair by glandular progenitors in mice and ferrets in vivo (8, 9, 24, 25).

### Generation and gross phenotype of Krt14 and Krt15 KO mouse models.

To establish a functional link between BC keratins and progenitor cell fate during regeneration, we generated Krt14 and Krt15 KO mice by removing exon 3 from Krt14 and Krt15 genes. Deletion of exon 3 causes a frameshift with a stop codon in exon 4, resulting in loss of exons 3–8 in both keratins. Some of the Krt15-KO mice that were older that 8 weeks had patchy hair loss (Supplemental Figure 5, E and F) but were otherwise phenotypically normal, with similar BW compared with their heterozygous littermates (Supplemental Figure 5H). By contrast, Krt14-KO mice had significantly lower BW, visually smaller lungs compared with those of their heterozygous littermates, frequent unilateral and bilateral ear defects, and increased mortality before and after weaning (Supplemental Figure 5, I–K).

**Figure 3:**
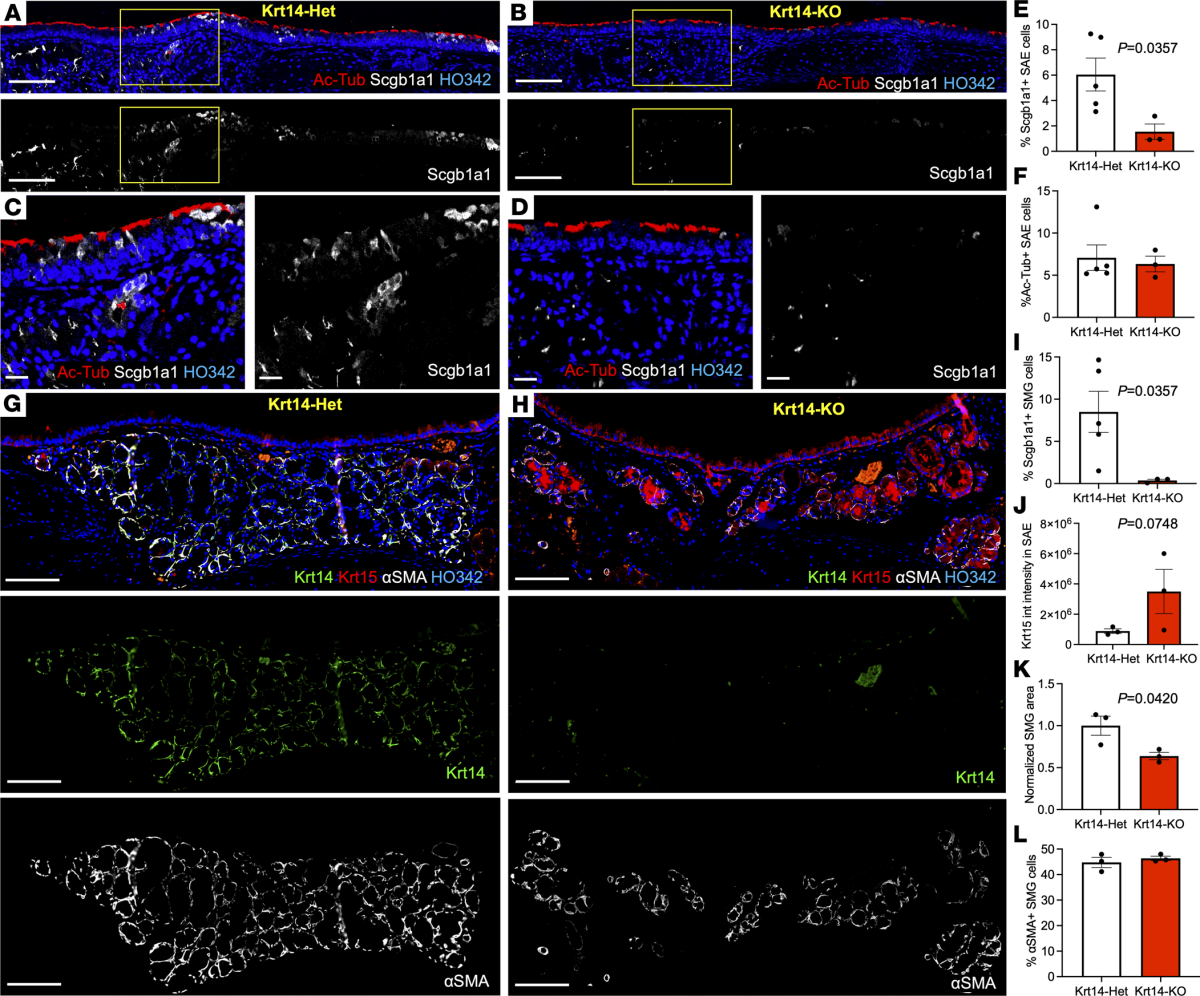
Krt14-KO mice have reduced glandular size, Scgb1a1 gland secretions, and abundance of club cells. (**A** and **B**) Immunofluorescence staining of 8-week-old Krt14-Het (**A** and **C**) or Krt14-KO (**B** and **D**) mouse tracheas for acetylated tubulin (Ac-Tubulin) and Scgb1a1. (**E** and **F**) Quantification of club cell (**E**) and ciliated cell (**F**) abundance in the SAE. (**G** and **H**) Immunofluorescence staining of 8-week-old Krt14-Het (**G**) or Krt14-KO (**H**) mouse tracheas for Krt14, Krt15, and α-SMA. (**I**) Quantification of Scgb1a1^+^ SMG cells. (**J**) Quantification of normalized integrated intensity of Krt15 staining in the SAE. (**K**) Quantification of the SMG size between Krt14-Het and Krt14-KO normalized to BW. (**L**) Quantification of percentage of α-SMA^+^ cells in the SMGs of Krt14-KO and Krt14-Het mice. Graphs show mean data ± SEM; *n* ≥ 3 independent mice. Statistical significance was determined by 2-tailed *t* test. Scale bars: 50 μm in **A** and **B,** 20 μm in **C** and **D**, and 200 μm in **E** and **F**.

**Figure 4:**
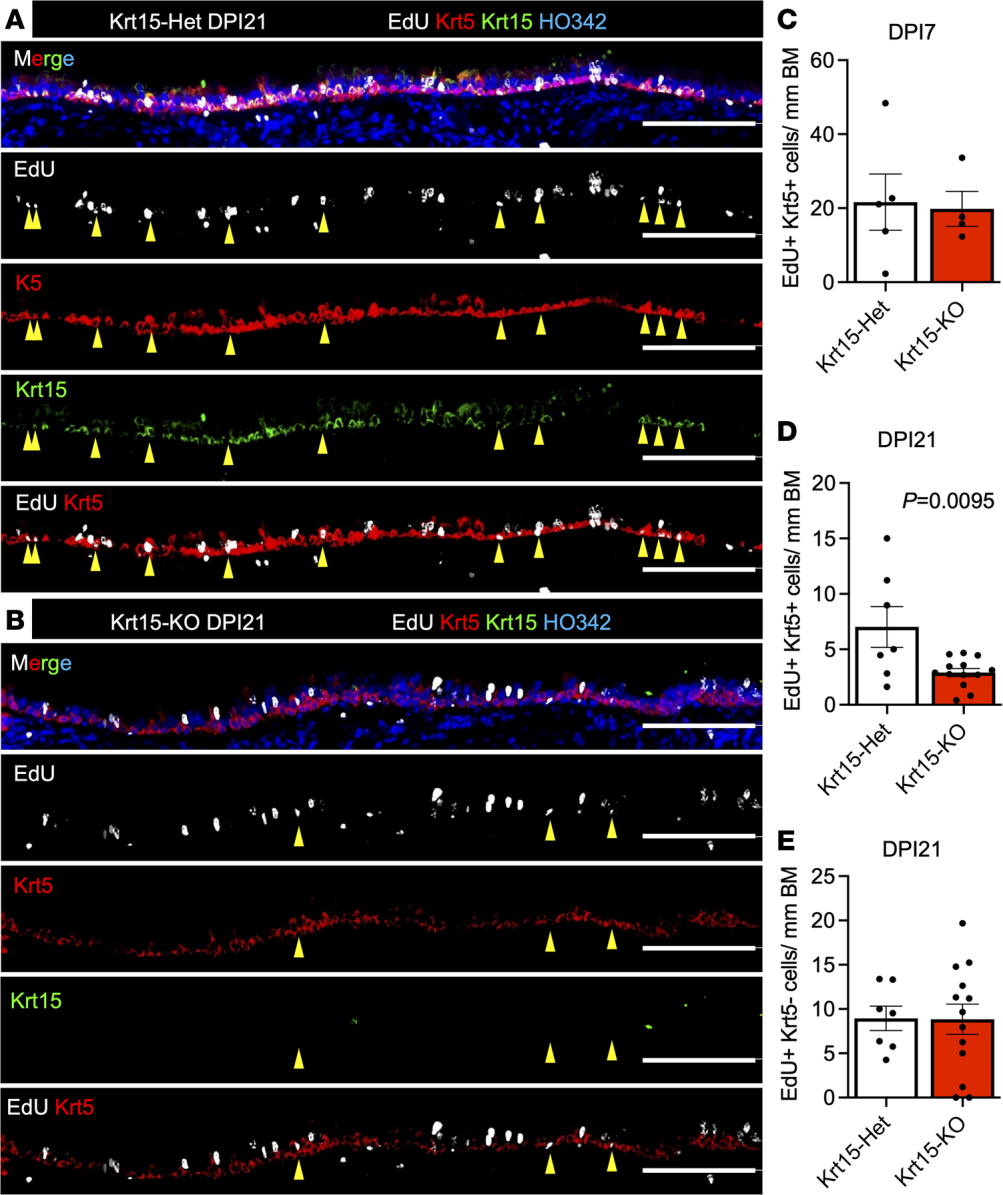
Krt15-KO leads to a decline in label-retaining BCs 21 days after injury. (**A** and **B**) Krt15-Het or Krt15-KO mice (5 weeks old) were injured with 250 mg/kg naphthalene and pulsed with EdU daily for the first 3 days immediately after injury. Immunofluorescence staining of the tracheas from Krt15-Het (**A**) or Krt15-KO (**B**) mice 21 days after injury. Yellow arrows indicate Krt5^+^ EdU^+^ cells. (**C**) The overall abundance of EdU^+^/Krt5^+^ cells in the SAE was quantified on DPI 7. (**D** and **E**) Abundance of EdU^+^ Krt5^+^ cells (**D**) and EdU^+^ Krt5^–^ cells (**E**) was quantified for DPI 21. Graphs show mean data ± SEM, *n* ≥ 4 independent mice. Significance was determined by 2-tailed *t* test. Scale bars: 100 μm.

Relatively few Krt14-KO mice survived weaning, and all of these animals were used for immunolocalization studies. Immunolocalization of Krt15 and Krt14 expression in the trachea confirmed its absence in Krt15-KO and Krt14-KO mice, respectively (Figure 3F and Figure 4B, and Supplemental Figure 5, A and B), suggesting that the mutant transcripts are most likely degraded by nonsense-mediated decay. We additionally validated Krt15 KO using primary airway BCs at passage 0. Krt15 heterozygous mice had Krt15 and Krt14 co-expression on day 3 in culture, at passage 0. As expected, we observed no filamentous staining for Krt15 and increased intensity of Krt14 staining in Krt15-KO cells, which was additionally confirmed by Western blot (Supplemental Figure 5, C, D, and G). These studies could not be performed on Krt14-KO BCs, because of poor survival of the mice.

### Evaluation of the airway phenotype of Krt14-KO and Krt15-KO mice.

Further examination of tracheas from Krt14-KO mice revealed the presence of ciliated cells, but fewer club cells, on the airway surface (Figure 3, A–F). SMGs of Krt14-KO mice, where Krt14 is normally expressed at homeostasis, expressed less Scgb1a1 than the glands of Krt14 heterozygous (Krt14-Het) mice (Figure 3, C, D, and I) and were smaller (when normalized by the mouse BW) (Figure 3, G, H, and K), but had a similar percentage of α-SMA^+^ MECs in the glands as Krt14-Het mice (Figure 3L). Overall, the tracheal surface epithelium of Krt14-KO mice had a trend toward higher levels of Krt15 compared with their Krt14-Het littermates (Figure 3, G, H, and J). By contrast, Krt15-KO and Krt15-Het mice had no reduction in gland size (Supplemental Figure 5, A and B) and abundance of ciliated and club cells (Supplemental Figure 6, A–E) similarly to that of Krt15-KO air–liquid interface (ALI) cultures in vitro (Supplemental Figure 6, F and G).

We next sought to evaluate the airway epithelial regenerative responses in Krt15-KO mice after a naphthalene injury. Because Krt15 expression and proliferative capacity of BCs both decline in human and ferret BO, we hypothesized that Krt15-KO mice would have fewer label-retaining BCs after injury. To test this, we subjected Krt15-KO and Krt15-Het mice to a moderate naphthalene injury (250 mg/kg) and pulsed them with EdU on DPI 3. We observed that Krt15-KO and Krt15-Het mice had a decreased abundance of Krt5^+^/EdU^+^ cells on DPI 21 but not on DPI 7 (Figure 4, A–D), suggesting a potential defect in basal stem cell maintenance and/or retention after injury. The abundance of EdU^+^ non-BCs (Krt5–) was not different between genotypes (Figure 4E).

### Krt15 and Krt14 Crispr KO.

**Figure 5:**
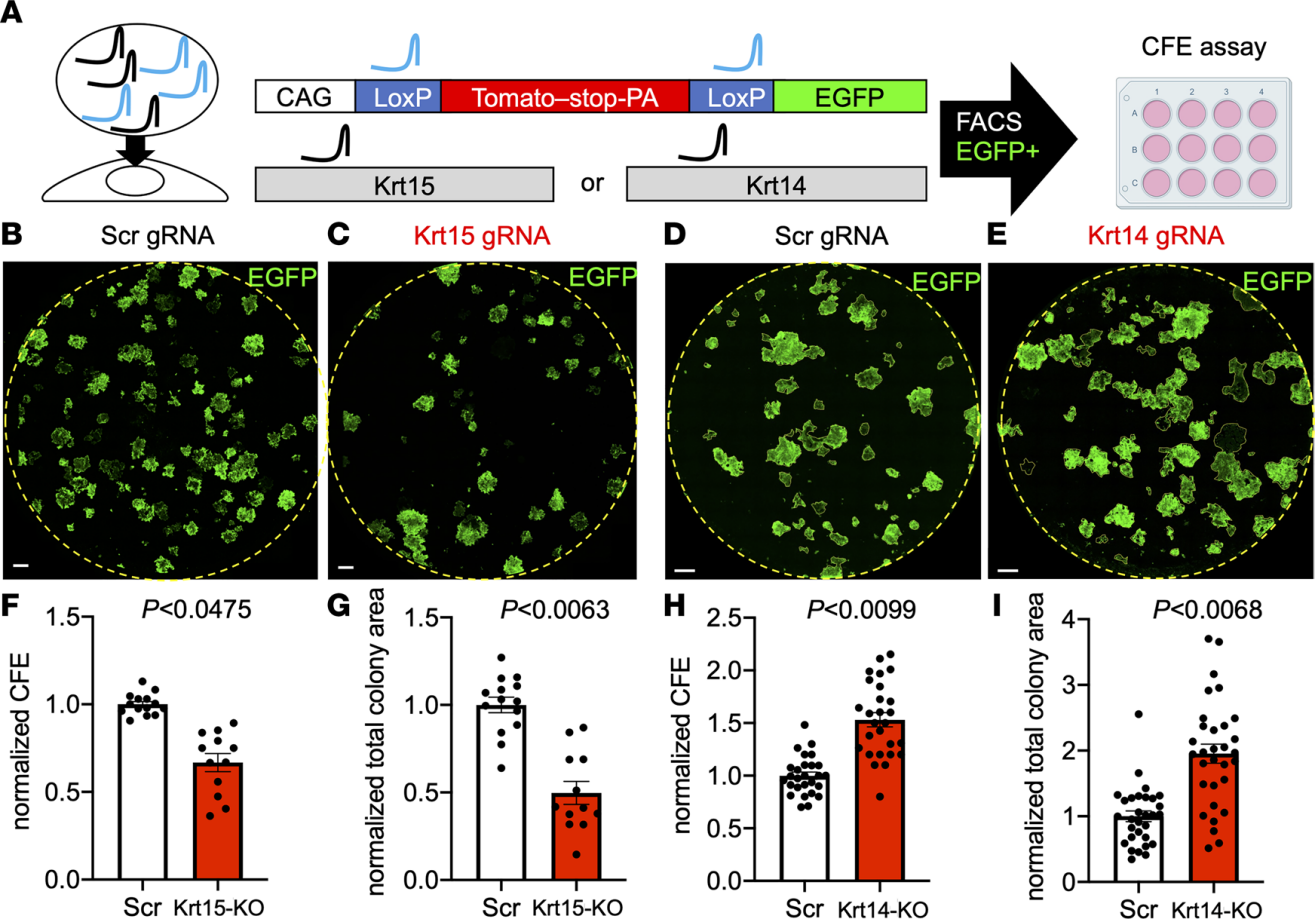
KOs of Krt15 and Krt14 have the opposite effects on clonogenic potential of primary SAE in vitro. (**A**) The outline of CRISPR KO experimental design. Passage-2 mouse SAE cells were used for Krt15-KO; passage-5 cells were used for Krt14-KO. Cells from ROSA-TG:H11-Cas9 mice were cotransfected with a 50:50 mix of LoxP and Krt15 or Krt14 gRNAs and, 7 days later, EGFP^+^ cells were sorted and seeded for CFE assay (500 cells/well of a 12-well plate). (**B**–**E**) Wells were fixed, stained, and imaged 7 days after the start of the CFE assay. (**F** and **H**) CFE was estimated by counting colonies with more than 50 cells in them. (**G** and **I**) Total colony area was calculated in ImageJ. Graphs show mean data ± SEM; *n* ≥ 3 independent transfections. Dots represent different technical replicates (i.e., different wells). Significance was determined by mixed-effect model nested 2-tailed *t* test. Scale bars: 1,000 μm. Scrambled (Scr) gRNAs were used as controls.

To study the functional significance of Krt15 loss, we performed Crispr KO in passage-2 primary airway BCs, most of which were still expressing Krt15. For Krt14-KO experiments, we used passage-5 cells, which had strong ubiquitous expression of Krt14 and relatively weak Krt15 expression by immunofluorescence staining (Figure 1F). We used the cells from transgenic mice that express Cas9 from the H11 locus (H11-Cas9) and the ROSA26-LoxP-Tomato-LoxP-EGFP reporter (ROSA-TG) to rapidly KO Krt15 or Krt14 on a cell population level after a gRNA transfection. We cotransfected passage-2 or passage-5 cells using a mix of gRNAs against LoxP and Krt15 or Krt14, respectively. We then performed FACS to collect EGFP^+^ cells, under an assumption that the reporter conversion from Tomato to EGFP would correlate with disruption of the Krt15 or Krt14 loci (Figure 5A). We verified Krt14-KO efficiency by Western blot (Supplemental Figure 7A), quantitative PCR (qPCR) (Supplemental Figure 7B), immunofluorescence staining (Supplemental Figure 7C), and sequencing of the Krt14 gene locus (Supplemental Figure 7, D and E), which had 92% of alleles knocked out. In some instances, we noticed re-expression of Krt15 in advanced-passage Krt14-KO cells (Supplemental Figure 8, A and B). Similarly, we verified Krt15-KO efficiency by immunofluorescence staining (Supplemental Figure 9A) and sequencing of the Krt15 gene locus (Supplemental Figure 9B), which had 93% of alleles with frameshift-containing insertions or deletions.

Immediately after FACS, 500 cells/well were seeded in a colony-formation efficiency (CFE) assay on irradiated 3T3-J2 fibroblasts. We observed that Krt15-KO cells formed fewer colonies that were also smaller compared with the scrambled gRNA–treated control cells (Figure 5, B, C, F, and G), suggesting that loss of Krt15 is detrimental for BC proliferative capacity. A similar decline in proliferative capacity was observed in the primary cells from ferrets that develop BO (14). In this work, we demonstrated that BCs of ferrets with BO and RAS had elevated levels Krt14 and decreased Krt15 in their SAE (Figure 1, A–D). In contrast, Krt14-KO cells formed more colonies that were larger compared with the scrambled gRNA–treated controls (Figure 5, D, E, H, and I). Overall, loss of Krt15 or Krt14 led to the opposite outcomes in terms of proliferative capacity. Krt15-KO led to a decline in clonogenicity and resulted in a basal keratin profile similar to that of chronic injury and BO (Krt14^+^/Krt15^–^), whereas Krt14-KO improved clonogenicity and resulted in a basal keratin profile similar to that of healthy homeostatic airways (Krt15^+^/Krt14^–^).

### Nuclear elongation in differentiating Krt14-KO BCs.

**Figure 6:**
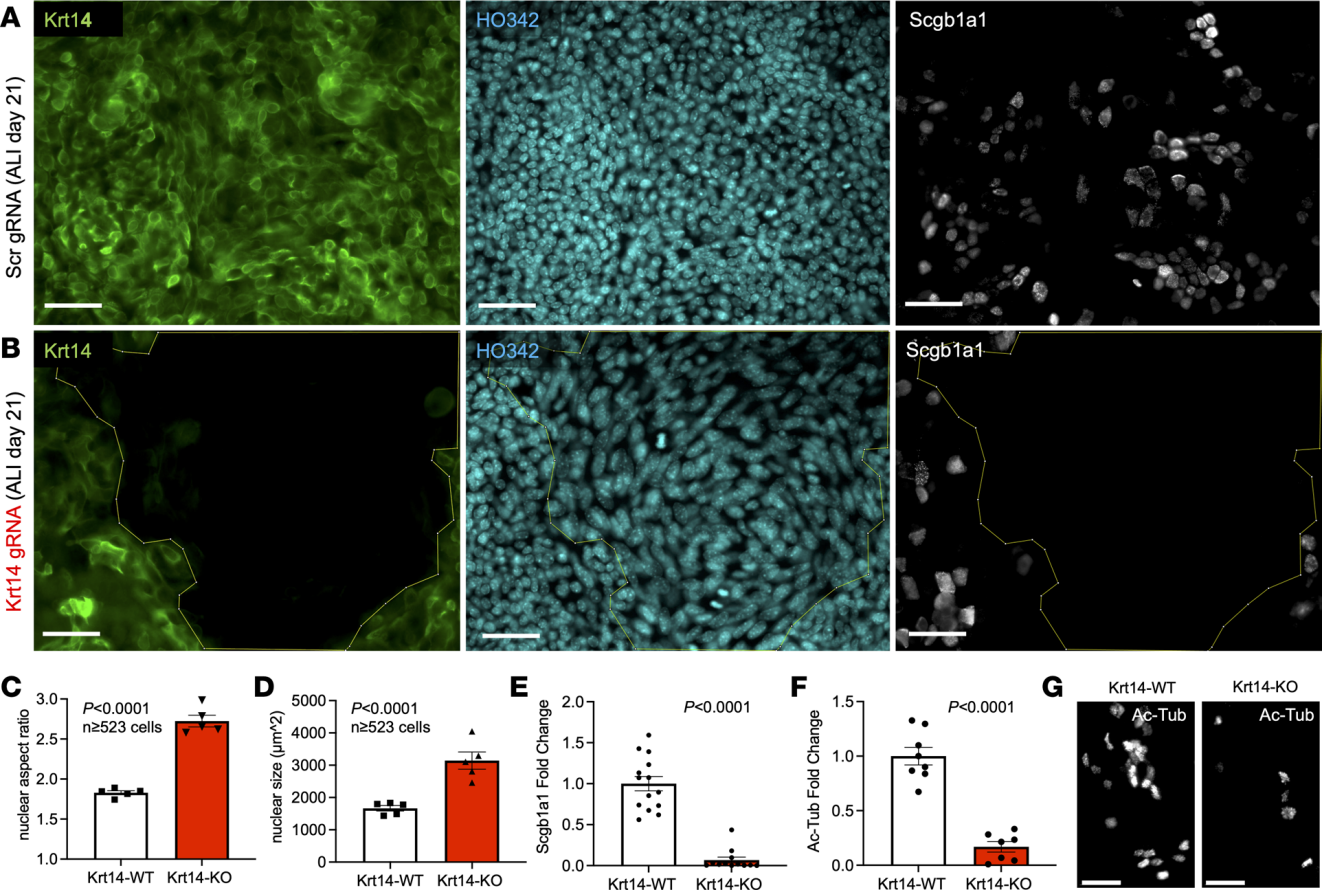
Differentiating Krt14-KO cells have enlarged and elongated nuclei and impaired club and ciliated cell specification. Passage 5 ROSA-TG:H11-Cas9 cells were transfected with a mix of Krt14 and Tomato gRNAs, after 7 days sorted for Tomato-/EGFP- cells and grown on ALI for 21 days. (**A** and **B**) Immunofluorescence staining for K14, nuclei (HO342) and Scgb1a1 was performed *en face* on day 21 cultures. (**C** and **D**) Nuclear aspect ratio and size were quantified in Metamorph from cells grown on the ALI for 21 days. (**E** and **F**) Fold change in of Scgb1a1 and acetylated tubulin (Ac-Tub) area staining was quantified using ImageJ. (**G**) Representative confocal micrographs of Krt14-KO and WT cultures on ALI day 21. Graphs shows mean ± SEM, *n* ≥ 5 transwells. Graphs in **E** and **F** show *n* ≥ 3 experiments pulled, graph in **C** is a representative of *n* = 2 experiments and the graph in **D** shows *n* = 1 experiment (cells collected from 3-4 mice were used per experiment). Significance was determined by 2-tailed *t* test in **C** and **D** and by mixed effects model Nested 2-tailed *t* test in **E** and **F**. Scale bars: 50 μm. Scrambled (Scr) gRNA was used as a control.

We also evaluated the ability of BC progenitor cells to differentiate at an ALI. To approach this question and allow for more flexibility in immunostaining with cell-specific markers, we cotransfected BCs with gRNAs against Tomato and Krt14 and subsequently sorted for nonfluorescent cells. We observed that the areas of culture lacking Krt14 effectively had no Scgb1a1^+^ club cells, compared with Krt14^+^ regions of the culture (Figure 6, A, B, and E). Thus, Krt14 appears to have cell-autonomous BC functions in club cell differentiation. We additionally noticed a difference in nuclear size and shape in Krt14-KO cells compared with Krt14^+^ cells during differentiation (Figure 6, A–D). Overall, the abundance of club and ciliated cells was significantly decreased in Krt14-KO cultures (Figure 6, F and G, and Supplemental Figure 10, A and B). Nuclear shape and differentiation of Krt15-KO BCs into ciliated and club cells was unaltered (Supplemental Figure 6, F and G).

### Krt14 and Krt15 selectively influence migration of SMG cells as compared with SAE cells.

To further define the roles of Krt14 and 15, we tested how the loss of each keratin affected cells migration in vitro. To simulate migration of SMG progenitors through the glandular ducts onto the airway surface, we performed a transwell migration assay in which cells migrate through 8 μm pores toward TGF-β1, a chemoattractant (Supplemental Figure 11A). We observed that migration of Krt15-KO and Krt15-Het SAE was similar, whereas the Krt15-KO SMG cells migrated more efficiently than the Krt15-Het controls (Supplemental Figure 11, B and C). We also tested Krt14-KO and WT cells in a competitive migration assay in which we seeded Krt14-KO and WT cells at approximately a 1:1 ratio and stained the underside of the transwell after 12 hours to record the new Krt14-KO to WT ratio in the migrated cell population (Supplemental Figure 11D).

We observed that the KO to WT ratio shifted in favor of Krt14-KO in migrated SMG cells compared with the input ratio, but it remained similar in the migrated SAE population (Supplemental Figure 11, E and F). Overall, Krt14-KO and Krt15-KO in SMG, but not in the SAE cells, facilitated migration in vitro. The observed effects might be explained by decreased cellular and nuclear rigidity in the absence of Krt14^+^ and Krt15^+^ IFs in the KO SMG cells.

### Krt14-KO airway BCs lose Sfn and upregulate p63, whereas Krt15-KO cells retain Sfn and downregulate p63 early in differentiation.

To understand the mechanism by which Krt14 and Krt15 differentially influence the fate of BCs, we looked at the key biochemical differences between these 2 structurally similar keratins. One notable residue was cysteine 373, which is conserved in Krt14 but is replaced by alanine in Krt15. The importance of this residue for proliferation and differentiation of keratinocytes was elegantly described by Guo et al. (15). Using a proteomics screen, those authors discovered Sfn was the top Krt14 interacting protein. They demonstrated that proper intracellular distribution of Sfn depends on C373 of Krt14 in vivo.

**Figure 7:**
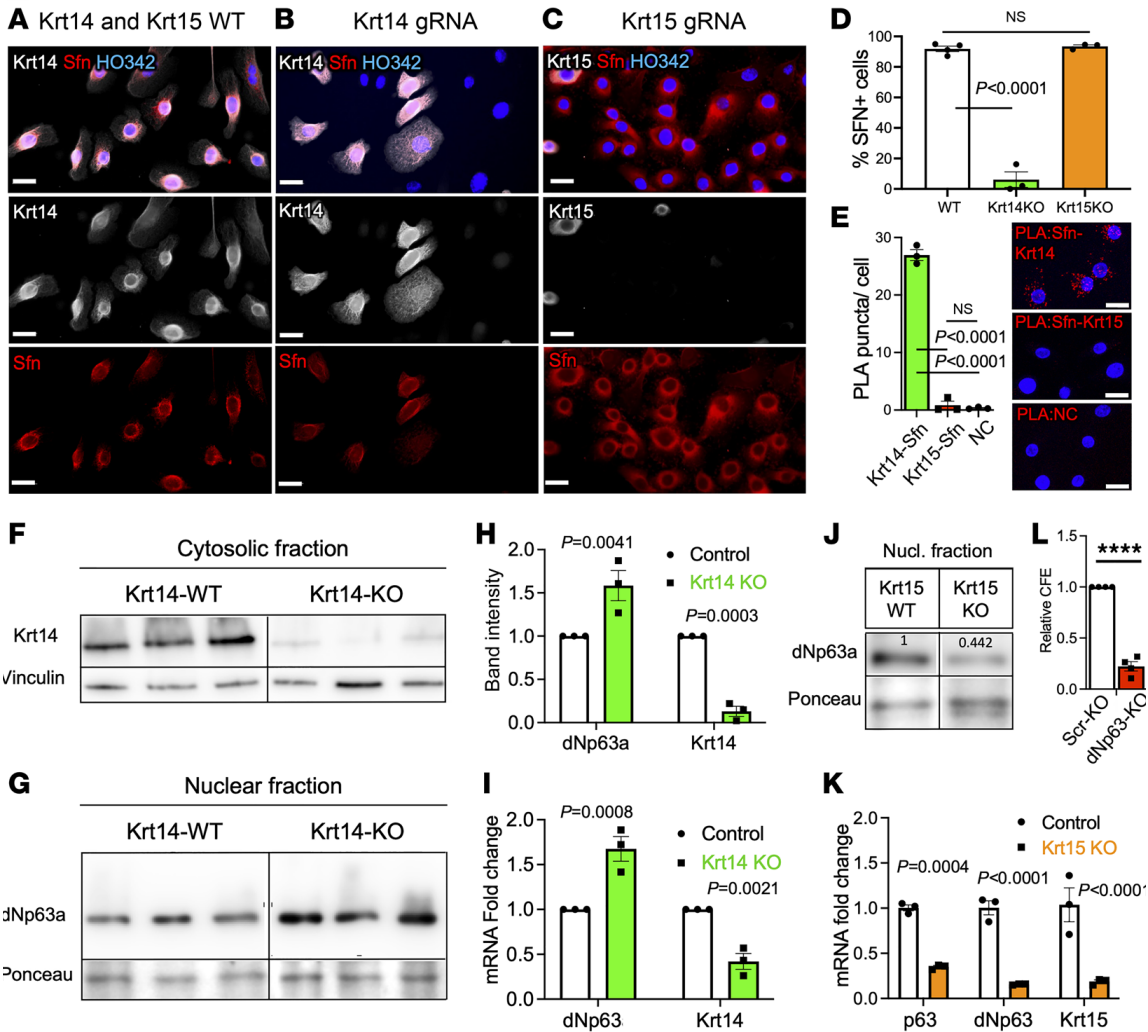
Krt14-KO airway BCs lose Sfn and upregulate p63, whereas Krt15-KO cells downregulate p63 early in differentiation. (**A**–**C**) Immunofluorescence staining of Krt14 and Sfn in Krt14-WT (**A**), Krt14-gRNA–treated cells (**B**) and Krt15-gRNA–treated primary airway BCs (**C**). (**D**) Quantification of immunofluorescence staining for Sfn from panels **A**–**C**. (**E**) Proximity ligation assay (PLA) of the interaction between Krt14 or Krt15 and Sfn or negative control (NC) in passage 2 (p2) healthy human SAE and quantification of the PLA signal. (**F** and **G**) Western blot analysis of the cytosolic (**F**) and nuclear (nucl) (**G**) fractions collected from Krt14-WT and Krt14-KO airway BCs on ALI day 5. (**H**) Normalized quantification of Western blot band intensity. (**I**) qPCR analysis of Krt14-KO and Krt14-WT differentiating cultures on ALI day 5. (**J**) Western blot analysis of the nuclear fraction collected from Krt15-KO airway BCs on ALI day 5. (**K**) qPCR analysis of Krt15-KO and Krt15-WT differentiating cultures on ALI day 5. (**L**) Quantification of CFE of dNp63-KO compared with Scrambled-KO primary mouse SAE cells. Crispr-KO and CFE assays were performed similarly to Krt15-KO in Figure 5. (**E**, **H**, **I**, and **L**) Graphs show mean data ± SEM from 3 independent pools of primary cells. (**K**) Graph mean data ± SEM of technical replicates from a cell pool isolated from 3 independent Krt15-WT or KO mice. (**F**, **G**, and **J**) Lanes were run on the same gel but were noncontiguous. Statistical significance in was determined by 2-way ANOVA, Sidak multiple comparison test (**H**, **I**, and **K**); by 1-way ANOVA, Tukey multiple comparison test (**D** and **E**); or by 2-tailed *t* test in **L**; ****P < 0.0001). Scale bars: 20 μm.

Sfn and p63 play opposing roles in epidermal tumorigenesis, and Sfn heterozygous mice have increased p63 levels in their hyperproliferative epidermis (26, 27). This could be due to dNp63a being targeted for proteasomal degradation by Sfn (28). Therefore, we tested the hypothesis that Sfn abundance and/or distribution would be affected by the loss of Krt14 and whether it would result in p63 upregulation. We observed a marked decrease in Sfn staining in Krt14-KO, but not in Krt15-KO, BCs (Figure 7, A–D). We demonstrated that Sfn interacts with Krt14, but not with Krt15, using proximity ligation assay (Figure 7E). Additionally, we observed elevated nuclear p63 protein abundance as well as increased abundance of p63 transcripts in Krt14-KO cultures early in differentiation (Figure 7, F–I). The effect on p63 was the opposite in Krt15-KO mice, in which we observed a reduction of p63 transcript and protein abundance (Figure 7, J and K). Functionally, we performed a Crispr-KO of dNp63 in primary mouse SAE and observed a reduction in clonogenicity compared with the scrambled control (Figure 7L), which was similar to Krt15-KO. This finding suggests that unlike Krt15-KO, Krt14-KO SAE might favor prolonged proliferation over differentiation through a mechanism involving downregulation of the tumor-suppressor Sfn and upregulation of an oncogene p63.

## Discussion

We show that Krt14 and Krt15 expression in surface airway BCs functionally affects proliferative potential and differentiation. Because Krt14 is upregulated in the SAE after injury, it likely demarcates transit-amplifying cells (8, 10–14). Transit-amplifying cells have a limited capacity to replicate and are primed to differentiate after a limited number of divisions (29). We observed that Krt14-KO primary airway BCs have increased clonogenic capacity (Figure 3) and impaired club and ciliated cell differentiation (Figures 4 and 6). Therefore, Krt14 appears necessary to prime airway BCs for differentiation by limiting their replicative potential as progenitors. Krt14’s counterpart, Krt15, is expressed in homeostatic Krt5^+^ BCs, which serve as stem cells (6). Krt15-KO early-passage primary BC cultures acquire the keratin profile similar to that of exhausted progenitors in CLAD (Krt14^+^/Krt15^–^). Freshly harvested primary progenitors from ferrets that developed CLAD showed a decreased clonogenic potential in CFE assays (14), which is similar to what we observed in Krt15-KO cells. Additionally, airway BCs in CLAD show decreased p63 abundance (14), which is similar to what we saw in differentiating Krt15-KO cells and opposite of that in Krt14-KO cells. Overall, Krt14-KO cells resemble the keratin profile of homeostatic airway BCs (Krt15^+^/Krt14^–^), which had the highest clonogenic potential in CFE assays by Swatek et al. (14), similar to Krt14-KO CFE assays in this study.

Prior in vitro Krt14 loss-of-function studies primarily used squamous epithelial cell lines, like HaCaT and AW13516 (30, 31). These studies demonstrated that knockdown of Krt14 results in defects in both proliferation and differentiation. Our data on Krt14-KO in primary airway BCs do not show decreased proliferation; instead, they show enhanced proliferative capacity in the absence of Krt14. It is important to consider that genetic compensation by other keratins (e.g., Krt15) is triggered by nonsense-mediated decay and would only occur in a KO, but not in a knockdown, which could explain the discrepancy. The observed difference in phenotypes can also suggest that Krt14 has context-dependent functions in different types of epithelia.

In humans and mice, frameshift-inducing Krt14 mutations cause epidermolysis bullosa simplex, a disease characterized by skin blistering that can also be caused by mutations in Plectin (32–35). Elongation and enlargement of the nuclei that we observed in Krt14-KO BCs during their differentiation (Figure 6) closely resemble the phenotype of Plectin KO cells (36). Plectin binds keratin IFs and attaches them to nucleoskeleton through the LINC complex as well as linking the filaments with one another and with integrins that mediate cells’ attachment to extracellular matrix (37, 38). The origin of altered nuclear shape in Krt14-KO BCs might be due to compromised attachment of IFs to nucleoskeleton by Plectin. Altered nuclear size and shape can affect trafficking of Yap, which might explain the increased proliferative capacity and impaired club and ciliated cell differentiation of Krt14-KO BCs observed in our system. Specifically, Elosegui-Artola et al. (39) demonstrated that elongated nuclei favored nuclear import of Yap, a pro-proliferation transcription factor. Work is needed to further elucidate this relationship in our system.

Previous reports have indicated perinatal lethality in Krt14-KO mice lacking exon 1, but some of the animals were able to survive after weaning age, when their epidermal phenotype became less severe (33). Even though the study authors, Lloyd et al., demonstrated that Krt15 can heterodimerize with Krt5 and form IFs, Krt15 levels did not increase in their KO model. In contrast to that study, the Krt14-KO mice in our study had an overall less severe phenotype, with an apparent increase in Krt15 staining intensity in Krt14-KO mouse trachea (Figure 6). This may be due to genetic compensation for the loss of Krt14 by upregulation of Krt15. Unlike the previously described Krt14-KO mouse model in which the authors removed exon 1 of Krt14 (33), our model is subject to nonsense-mediated decay because we removed exon 3 of Krt14 and, therefore, the transcriptional initiation site was intact. Notably, rare human cases of Krt14 loss-of-function mutations are compensated for by upregulation of Krt15 and display a much less severe phenotype than that of the Krt14-KO mice characterized by Lloyd et al. (40, 41). Recent reports have described a mechanism whereby nonsense-mediated decay can lead to transcriptional compensation by closely related gene homologs exhibiting sequence similarity with the mutated gene’s mRNA (42). This mechanism may explain the findings of enhanced Krt15 expression in Krt14-KO BCs.

We generated Krt15 whole-body KO mice by deleting exon 3 (the same way as for Krt14-KO mice). These mice were viable and fertile and had a similar BW as their heterozygous littermates. We noticed that approximately half of the adult Krt15-KO mice (after 8 weeks of age) had partial hair loss on their flanks, head, back, or chest (Supplemental Figure 5). This can be attributed to a defect in maintenance of follicular stem cells, which are normally marked by Krt15 (43). Krt15 lineage tracing and genetic ablation studies using Krt15-Cre and inducible diphtheria toxin receptor mice have been carried out in the intestine and esophagus. These works reveal that Krt15 marks a radiation-resistant population of long-lived progenitors with enhanced clonogenicity (44, 45). The finding is in line with our observation that Krt15-KO airway basal stem cells have a decreased clonogenicity in vitro and decreased long-term label retention in airway BCs after injury in vivo (Figures 4 and 5).

The recent article by Guo et al., in which the researchers looked at keratinocyte proliferation and differentiation in response to C373A mutation in Krt14, has partly informed the hypothesis in our model system. The Coulombe group observed enhanced proliferation, impaired differentiation, altered nuclear shape, and increased instances of nuclear fragmentation in the epidermis of Krt14C373A-mutant mice (15). This phenotype was similar to our own observations in Krt14-KO airway BCs, which had enhanced clonogenicity, impaired club and ciliated cell differentiation, and abnormal nuclear shape and size during differentiation. We propose that Krt15, which has alanine in place of cysteine 373, compensates for the loss of Krt14 in our system and subsequently resembles the C373A-mutant Krt14 that the Coulombe group used in their study (15).

Using a proteomics screen, Guo et al. identified 14-3-3 family members as the most abundant interactors with Krt14, in particular, Sfn was the most abundant K14 binding protein, and it had a much weaker interaction with C373A-mutant Krt14 compared with the WT. Consistent with these findings, we observed a robust protein–protein interaction between Krt14 and Sfn, but not between Krt15 and Sfn (Figure 7), which is logical considering the equivalent residue to C373 of Krt14 is alanine in Krt15. Guo et al. (15) demonstrated abnormal distribution of Sfn in Krt14 C373A-mutant epidermis, which they propose to be the reason for enhanced nuclear Yap localization and subsequent phenotypes of enhanced proliferation and impaired differentiation. We also observed a near-complete absence of Sfn in the Krt14-KO, but not in Krt15-KO, cells in the present study (Figure 7). Sfn functions as a tumor suppressor: its downregulation leads to immortalization of primary human keratinocytes by locking them in a stem cell–like state (46). Sfn has also been shown to target dNp63a for proteasomal degradation in response to DNA damage (28). Sfn heterozygous mice (+/Er) display hyperproliferative epidermis and increased nuclear p63 and Yap (27). Increased p63 expression can shift the balance in favor of prolonged proliferation, instead of differentiation, by repressing Notch signaling (26, 47–49), which is a pathway that specifies club cells in the airways (50). Consistent with reports in the literature, we observed enhanced proliferative capacity, impaired differentiation, decreased Sfn protein abundance, and increased p63 protein abundance after a KO of Krt14 in airway BCs. By contrast Krt15-KO resulted in impaired proliferative capacity, increased Krt14 expression, and decreased p63 expression, which, overall, resembles the BC’s phenotype in BO (14).

Our lead working model postulates that changes in Krt14/Krt15 content alters BC behavior by modifying Sfn abundance, which, in turn, affects signal transduction cascades that balance proliferation and differentiation (i.e., dNp63 nuclear export and proteasomal degradation). Notably, we observed increased dNp63 protein and mRNA levels in Krt14-KO mice, which cannot be solely explained by impaired degradation of dNp63 protein. However, dNp63 nuclear export is not the only way Sfn can mediate its role as a tumor suppressor. For example, Sfn has also been show to enhance p53 activity (51), which can further suppress dNp63 transcriptionally (52). Therefore, Krt14-induced changes in dNp63 transcript levels could be indirectly affected by Sfn action on p53.

### Conclusions.

Overall, this work offers insight into the functional significance of Krt14/Krt15 switching in airway BCs in BO and after injury and provides evidence that keratin composition can affect the fate of airway progenitors. Because this work does not exhaustively prove certain mechanistic details of our proposed working model, further exploration is needed on the molecular networks regulated by Krt14 from Krt15 and how these networks divergently influence BC behavior. Such studies have the potential to uncover novel pathways for BC reprogramming in the context of chronic lung disease states. Limits on passaging Krt14-KO primary BCs currently impose experimental limitations (e.g., Krt14 rescue experiments), thus future mechanistic studies would greatly benefit from a conditional KO approach by enabling temporal regulation of each keratin during BC proliferation and differentiation. Such conditional KO models would also facilitate interrogation of Krt14 and Krt15 functions during airway injury and repair in the absence of developmental compensation.

The findings we provide in this article show how the changes in Krt14 and Krt15 expression can influence the fate and behavior of airway BCs in vitro and in vivo, with some insights into the potential mechanisms driving these phenotypes. This study is limited in its ability to confirm a cause-and-effect relationship between the injury-associated basal keratin changes and airway fibrosis in BO, but it lays the groundwork for testing these relationships in the future. In this study, we relied heavily on in vitro and murine models of Krt14-KO and Krt15-KO mice, which are insufficient to test the direct relevance to human BO because mice do not develop BO lesions in the injury models available to us. In the future, conditional Krt15-KO and Krt14-KO ferret lung transplantation models can be used to interrogate potential causative effects of Krt14 and Krt15 expression in BO. Nonetheless, our current findings provide insights into airway epithelial injury repair processes that are universally relevant to many airway pathologies.

## Methods

### Animal studies.

All animal studies were approved by the University of Iowa IACUC. The following strains of mice on a C57BL/6 background were used: B6J.129(Cg)-Igs2tm1.1(CAG-cas9*)Mmw/J (H11-Cas9); B6.129(Cg)-Gt(ROSA)26Sor^tm4(ACTB-tdTomato,-EGFP)Luo^/J (ROSA-TG); B6.129(Cg)-Krt15^Tm1ex3^ (Krt15-KO); and B6.129(Cg)-Krt14^Tm1ex3^ (Krt14-KO). See the Supplemental Methods for details on how the mice were injured with naphthalene, and pulsed with 5-ethynyl-2′-deoxyuridine.

### Primary cell isolation and culture.

Cells from the SAE were isolated from 3–6 mouse tracheas, using enzymatic digestion as described in Supplemental Materials and Methods. Cells were cultured in small airway growth medium (SAGM; Lonza), in F-medium (for CFE assays) or in PneumaCult ALI media (for ALI assays; Stemcell Technologies) (more details are provided in the Supplemental Materials and Methods).

### Crispr/Cas9 KO in vitro.

Crispr/Cas9 KOs were performed in primary SAE cells isolated from H11-Cas9:Rosa-TG mice. Cells were transfected with a 1:1 mixture of gRNAs against the reporter (LoxP or Tomato) and against the gene of interest (Krt14 or Krt15) using Lipofectamine RNAiMax (Thermo Fisher Scientific). Cells were fluorescently sorted, and the KO efficiency was determined as described in Supplemental Materials and Methods.

### Generation of Krt14 and Krt15 KO mice.

Krt14 and Krt15 KO mice were generated at the University of Iowa Genome Editing Core Facility by pronuclear injection of C57Bl6j zygotes with Crispr/Cas9 ribonucleoproteins (RNPs) containing gRNAs targeting intronic sequences around exon 3 of either Krt14 or Krt15 genes. The resulting chimeric F0 mice were backcrossed to WT C57Bl6j mice and the F1 male offspring were used in breeding with WT C57Bl6j mice after the confirmation of the KO by Sanger sequencing.

Pronuclear-stage embryos were collected in KSOM medium (Millipore; catalog MR101D) and injected with ribonucleoproteins. The embryos (*n* = 15–25) were immediately implanted into the oviducts of pseudo-pregnant female Institute of Cancer Research mice (Supplemental Materials and Methods).

### ALI differentiation and CFE assays.

Primary cells from H11-Cas9:Rosa-TG mice were transfected with the specified gRNA and fluorescently sorted prior to ALI differentiation and CFE assays.

For ALI assays, FACS-sorted cells were expanded in SAGM to obtain sufficient numbers and then seeded at a density of 3 × 10^5^ cells/well on a 0.33 cm^2^ polyester (0.45 μm pores) transwell membrane (Corning) in SAGM medium (Lonza) for at least 24 hours until confluency was reached. Cultures were then transitioned to an ALI (day 0), and SAGM medium was replaced with basolateral PneumaCult ALI (Stem Cell Technologies). Cells were fixed, stained, and imaged on the indicated days of the assay (Supplemental Materials and Methods).

For CFE assays, immediately after FACS, 500 cells were seeded onto a monolayer of irradiated 3T3-J2 fibroblasts in 12-well plates. Wells were fixed on day 7 after seeding and were stained, and imaged for quantification (Supplemental Materials and Methods).

### Ex vivo scratch assays and whole-mount ferret trachea staining.

WT adult ferret tracheas were dissected along the membranous side and scratched with a stiff nylon brush (2 mm diameter). Explants were then cultured in F-medium. On specified days, the tracheas were pulsed with 10 μM EdU (Thermo Fisher Scientific). On specified days, the explants were fixed, stained, cleared, and imaged en face as whole mounts (Supplemental Materials and Methods).

### Immunofluorescence.

Mouse tracheas were resected and fixed in 4% paraformaldehyde (PFA) for 2 hours, washed in PBS, and embedded in paraffin or in OCT frozen blocks. Longitudinal sections were cut, fixed in PFA for 20 minutes, and stained as described in the Supplemental Materials and Methods. EdU was detected using the Click-iT EdU Cell Proliferation Kit for Imaging (Thermo Fisher Scientific) according to the accompanying protocol, with an increased incubation time when performed on whole-mount ferret tracheas. A complete list of primary Abs used can be found in Supplemental Table 1.

### Western blot analysis.

Protein samples were fractionated using NE-PER kit (Thermo Fisher Scientific) following the provided protocol. Protein concentration was measured using the Pierce BCA assay kit (Thermo Fisher Scientific). Samples were run on an SDS PAGE gel under reducing conditions and transferred onto Amersham Protran 0.45 nitrocellulose membrane (GE Healthcare), which was subsequently probed using the specified Abs and imaged using an Ai600 imager (GE Healthcare). Band intensity was analyzed using ImageJ (NIH) and normalized to the loading control and to the WT control.

### qPCR analysis.

RNA was isolated from cells using an RNeasy kit (Qiagen) and converted into cDNA using a High-Capacity cDNA RT kit (Applied Biosystems) according to the manufacturer’s instructions. qPCR reactions were set up using Power SYBR (Applied Biosystems) and run on a CFX Connect Real-Time PCR detection system (Bio-Rad). The expression data were normalized using ΔΔ-CT method. See the Supplemental Materials and Methods for details and the primer sequences.

### Transwell migration assays.

Migration assays were performed using primary mouse SAE or SMG cells, which were seeded to confluency (3 × 10^5^ cells/well) into 804G-coated transwells (diameter, 6.5 mm, pore size, 8 μm; Corning, catalog 353097) in SAGM with TGF-β1 (10 ng/mL; PeproTech) in the bottom chamber, which served as a chemoattractant. Cells on the underside of the transwell were fixed and stained after 12 hours of migration. More details are provided in the Supplemental Materials and Methods.

### Proximity ligation assay.

The assay was carried out using a Duo Link PLA kit (MilliporeSigma, catalog DUO92202). The assay was carried out on permanox chamber slides (Lab-Tek, catalog 177445) according to the provided protocol. Primary Ab incubation was carried out overnight at 4°C. Experimental samples and a negative control were run on the same slides. See the Supplemental Materials and Methods for more details.

### Data availability.

The data sets generated and/or analyzed in the present study are available from the corresponding author on reasonable request.

### Statistics.

Results are reported as mean ± SEM; individual dots on the graphs in figures represent biological replicates unless stated otherwise in the figure legend. Statistical analysis was conducted using Prism, version 8 (GraphPad Software); *n* equaled the number of independent animals unless stated otherwise in figure legends. The statistical tests used are stated in each figure legend. Significant differences between 2 groups were assessed using a 2-tailed *t* test. Data were considered significant at *P* < 0.05.

### Study approval.

All procedures were approved by the University of Iowa IACUC and conducted according to the NIH’s *Guide for the Care and Use of Laboratory Animals* (National Academies Press, 2011). Collection of human samples was approved by the IRB at the University of Iowa.

## Author contributions

VI, JFE, and KRP designed the research studies. VI conducted experiments and acquired data. VI, TJL, KWF, CBG, AS, ACP, BAA, SP, JFE, and KRP analyzed data. VI wrote the manuscript. VI, JFE, KRP, KWF, and TJL edited the manuscript. All authors gave final approval and agree to be accountable for all aspects of the work.

## Supplementary Material

Supplemental data

## Version 1 12/13/2022

In-Press Preview

## Version 2 01/24/2023

Electronic publication
