# Supplementary material for: Krt14 and Krt15 differentially regulate regenerative properties and differentiation potential of airway basal cells
Source: JCI Insight. 2023 Jan 24;8(2):e162041. doi: 10.1172/jci.insight.162041 (PMC9977304; doi:10.1172/jci.insight.162041)
Supplement: Supplemental data [file jciinsight-8-162041-s154.pdf]

Supplemental Materials and Methods

Animal studies

All animal studies were approved by the University of Iowa IACUC (Institutional Animal Care and Use Committee). All animals were housed under specific pathogens-free conditions. Abbreviated names for transgenic mouse lines are listed below: B6J.129(Cg)-Igs2tm1.1(CAG-cas9*)Mmw/J (H11-Cas9), B6.129(Cg)-Gt(ROSA)26Sor$^{tm4(ACTB\text{-}tdTomato,\text{-}EGFP)Luo}$/J (ROSA-TG), B6.129(Cg)-K15^{Tm1ex3} (Krt15-KO), B6.129(Cg)-K14^{Tm1ex3} (Krt14-KO).

For naphthalene injury, mice were given a single i.p. injection of Naphthalene (250 mg/kg body weight) dissolved in corn oil; the control animals were injected with just corn oil. In specified instances, one i.p. injection of 5-ethynyl-2'-deoxyuridine (EdU) solution (500μl of 3 mg/mL EdU dissolved in 5% dextrose) was given following injury on day 3. For polidocanol injury, fully anesthetized ferrets were given 500μl of 1% polidocanol through a bronchoscope in the right and left lower lobes. Ferret lung transplantation was performed according to the previously described protocol (1), where left lower lobe was transplanted into a healthy adult wild-type ferret.

Transgenic animal generation

C57BL/6J mice were purchased from Jackson Labs (000664; Bar Harbor, ME). Male mice older than 8 weeks were used to breed with 3-5-week-old super-ovulated females to produce zygotes for pronuclear injection. Female ICR (Envigo; Hsc:ICR(CD-1)) mice were used as recipients for embryo transfer. All animals were maintained in a climate-controlled environment at 25°C and a 12/12 light/dark cycle. Animal care and procedures conformed to the standards of the Institutional Animal Care and Use Committee of the Office of Animal Resources at the University of Iowa.

Preparation of Cas9 RNPs and the injection mix: Chemically modified CRISPR-Cas9 crRNAs and CRISPR-Cas9 tracrRNA were purchased from IDT (Alt-R® CRISPR-Cas9 crRNA; Alt-R® CRISPR-Cas9 tracrRNA (Cat# 1072532)). The crRNAs and tracrRNA were suspended in T10E0.1 and combined to 1 ug/ul (~29.5 uM) final concentration in a 1:2 (ug:ug) ratio. The RNAs were heated at 98°C for 2 min and allowed to cool slowly to 20°C in a thermal cycler. The annealed cr:tracrRNAs were aliquoted to single-use tubes and stored at -80°C.

Cas9 nuclease was also purchased from IDT (Alt-R® S.p. HiFi Cas9 Nuclease). Cr:tracr:Cas9 ribonucleoprotein complexes were made by combining Cas9 protein and cr:tracrRNA in T10E0.1 (final concentrations: 300 ng/ul (~1.9 uM) Cas9 protein and 200 ng/ul (~5.9 uM) cr:tracrRNA). The Cas9 protein and annealed RNAs were incubated at 37C for 10 minutes. The RNP complexes were combined with double-stranded repair template (1966 bp (Krt14) or 1927 bp (Krt15) EcoRV/Sal1 fragments of target vector) and incubated an additional 5 minutes at 37C. The concentrations in the injection mix were 30 ng/ul (~0.2 uM) Cas9 protein and 10 ng/ul (~0.6 uM) each cr:tracrRNA and 5 ng/ul double-stranded repair template.

Collection of embryos and injection: Pronuclear-stage embryos were collected in KSOM media (Millipore; MR101D) and washed 3 times to remove cumulous cells. Cas9 RNPs and double-stranded repair template were injected into the pronuclei of the collected zygotes and incubated in KSOM with amino acids at 37°C under 5% CO_2 until all zygotes were injected. Fifteen to 25 embryos were immediately implanted into the oviducts of pseudo-pregnant ICR females.

The following gRNAs were used:

Krt14_5PA	AACGTGATGGCTTTCACACC
Krt14_Int_3_4_A	ATCCATCCACCCTCTTAAGC
Krt15_Int_2_3_B	TAAAGGGAGTCTACAGAAAG
Krt15_Int_3_4_C	GGGGATCCACCTGACACAAT

Primary cell isolation and culture

Primary Cell Isolation: Primary surface epithelial and submucosal gland cells were isolated from murine tracheas using enzymatic digestion. Tracheas from 3-6 mice were dissected longitudinally and digested in 3 mg/ml Pronase (Roche), which was dissolved in F12 media (Gibco), for 45 min at 37ºC on a rocker. This was done to dissociate surface airway epithelial (SAE) cells. Detached SAE cells were then separated from the remaining tissue by passing through the 100 μm strainer. Cells were then washed twice in excess of complete DMEM (DMEM with 10% FBS, 1% Penicillin/Streptomycin). The remaining tissue containing submucosal gland (SMG) cells was then incubated in 1X Collagenase/Hyaluronidase (STEMCELL Technologies, Inc.) dissolved in F12 for 20 min at 37ºC on a rocker. 1 ml of Trypsin/EDTA (0.025% Trypsin, 0.01% EDTA; Thermo Fisher Scientific) was then added to 3 ml of Collagenase/ Hyaluronidase solution for an additional 5-10 min at 37ºC. After the digestion detached SMG cells were passed through a 100 μm cell strainer and washed in an excess of complete DMEM before plating on 804G-coated plastic plates.

Primary Cell Culture: Primary cells were cultured under the conditions used to propagate airway basal cells (BCs) and submucosal gland (SMG) progenitors as described previously.(2) Airway stem cells were cultured in Small Airway Growth Medium (SAGM) (Lonza, Cat#CC-3118) with the added 10 μM Y-27632 (Tocris, Cat#1254), 1 μM DMH-1 (Tocris, Cat#4126), 1 μM A83-01 (Tocris, Cat#2939), and 1 μM CHIR 99021 (Tocris, Cat#4423) and 1% Penicillin/Streptomycin. 2-D cell cultures were grown on tissue culture-treated plastic pre-coated with filter-sterilized ECM-enriched 804G-conditioned media for at least 1h as previously described (2). For the air-liquid interface cultures (ALI), cells were seeded onto 0.45μm pore size polyester transwell membranes (Corning) which were pre-coated with 804G-conditioned media. After establishment of a tight monolayer, cultures were transitioned to air-liquid interface and maintained in basolateral

PneumaCult ALI media (STEMCELL Technologies, Inc.). Accutase (STEMCELL Technologies, Inc.) was used to detach the cells from culture dishes. During washes cells were pelleted for 5 minutes at 500g force.

Colony forming efficiency assays

We performed colony forming efficiency (CFE) assays on primary SAE BCs isolated from H11-Cas9:ROSA-TG mice. Cells edited with a specified gRNA and sorted using fluorescence activated cell sorting (FACS) immediately prior to the CFE assay. Cell counts from FACS were used to determine the starting seeding density. 24h prior to the start of the assays, we seeded a confluent monolayer of irradiated 3T3 fibroblasts (J2) into the wells of 12-well plates. Complete DMEM (with 10% FBS and 1% Penicillin/Streptomycin) was used to culture J2 fibroblasts. Airway basal cells were seeded onto the irradiated J2 fibroblasts at a density of 500 cells/well in F-media [3:1 (v/v) DMEM (Invitrogen) : F-12 Nutrient Mixture (Gibco), 5% FBS, 1% Penicillin/Streptomycin, 10 ng/mL epidermal growth factor (Invitrogen), 24 µg/mL adenine (Sigma-Aldrich), 5 µg/mL insulin (Sigma-Aldrich), 0.01 mg/mL Gentamicin, 0.1% amphotericin B, 0.4 µg/mL hydrocortisone (Sigma-Aldrich), 8.4 ng/mL cholera toxin (Sigma-Aldrich) and 10 µmol/L Y-27632 (Tocris)] as previously described (2). Cultures were fixed using 4% paraformaldehyde (PFA) for 20 minutes ~7 days after the start of the assay, and then were stained as specified, and imaged using Zeiss LSM 880 confocal microscope (Carl Zeiss, Germany). See below for details on immunofluorescent staining and quantification of CFE assays.

Crispr/Cas9 Knockout

Crispr/Cas9 knockouts were performed in primary SAE cells isolated from H11-Cas9: Rosa-TG mice. Cells were grown to 60-80% confluence in 6-well plates and transfected using 0.3 nMoles of gRNA against the LoxP or Tomato and 0.3 nMoles of gRNA against the gene of interest (See Table 2 for details). Transfection was done using 10µl of Lipofectamine RNAiMax. FACS

was performed 2-3 days after transfection with LoxP gRNA and 7-10 days after transfection with Tomato gRNA. DNA was isolated from sorted cells using DNeasy Blood and Tissue Kit (Qiagen) and the gene locus was amplified by PCR. PCR product was either sent for Sanger sequencing with a subsequent peak de-convolution using ICE analysis (Synthego), or was TOPO cloned into a plasmid vector with a subsequent sequencing of plasmid DNA from individual bacterial colonies. ICE analysis and TOPO cloning approaches showed nearly identical results; therefore, ICE analysis was the primary method for determining knockout efficiency.

Immunofluorescence

Freshly dissected murine tracheas were fixed in 4% PFA for at least 2h at room temperature (RT) or for at least 24h at 4ºC. Samples were paraffin embedded by University of Iowa Comparative Pathology Laboratories. 6 µm paraffin sections were 'baked' onto glass slides at 45ºC overnight. After de-paraffinization through xylenes and a stepwise EtOH gradient (100% → 0%), samples were 'boiled' in Sodium Citrate buffer (10mM $Na_3C_6H_9O_9$, 0.05% Tween 20, pH=6.0) using a pressure cooker for 5 min to retrieve antigens. Slides were then incubated in blocking buffer containing 20% normal donkey serum, 0.3% Triton X-100, and 1 mM $CaCl_2$ in PBS for 1h at RT. Primary antibodies (See Table S1 for details) were diluted in the diluent buffer (1% normal donkey serum, 0.3% Triton X-100, and 1 mM $CaCl_2$ in PBS) and incubated on the sample slides overnight at 4°C. Slides were washed in PBS 3 times for 5-10 min and incubated with secondary antibodies and Hoechst 33342 (Invitrogen) in diluent buffer for 2h at room temperature. EdU staining was done using Click-iT™ Plus EdU Cell Proliferation Kit for Imaging (ThermoFisher Scientific) according to the provided protocol. ProLong Diamond media (Invitrogen) was used to mount the coverslips on the samples.

Cultures at the assay endpoints (both CFE, ALI and monolayer cultures) were fixed in 4% PFA for 20 minutes at RT, and subsequently washed 3 times in PBS for 10 minutes each. Primary and secondary antibody incubations were performed as described above.

Zeiss LSM 880 line-scanning confocal microscope (Carl Zeiss, Germany) or a Leica DM6 B upright microscope (Leica Mircosystems, Inc., United States) were used to acquire fluorescent images.

Image analysis

Composited immunofluorescent tile scans were quantified using the Metamorph Software, specifically, the Multi Wavelength Cell Scoring Application Module (Molecular Devices, LLC). For the images of airway sections, areas of interest (for example the SAE) were first cropped out of the original image using ImageJ. Each experimental point for *in vivo* quantifications represents an independent animal with an average of 2191+/-210 SAE cells and 1858+/-130 SMG cells scored. Label-retaining basal cell scoring in Figure 7 was done manually in a blinded manner by 2 people, scoring using Metamorph was not feasible due to technical limitations of the software. Epithelial coverage of the explants was scored in ImageJ by dividing the area stained with epithelial markers (Krt5, Krt14, Krt15) over the total explant area. Colony number for CFE assays was scored manually, while staining area in CFE and ALI was quantified automatically after setting an appropriate threshold in ImageJ.

Ex vivo scratch assays and whole mount ferret trachea staining

Tracheas from adult wild-type ferrets were used in ex vivo scratch assays. Freshly excised tracheas were cut open along the membranous side and brushed with a stiff nylon brush (Justman Brush Company, cat#415140, d=2 mm) along the proximal-distal axis. The explants were subsequently cultured in F-media and pulsed with 10 μM EdU (5-ethynyl-2'-deoxyuridine, ThermoFisher Scientific C10340) on day-3 post-injury (unless specified otherwise). The tissues were fixed on specified days post injury for 2-4h in 4% PFA at RT with agitation, then washed in PBS 3 times for 15 min and incubated in Sodium Citrate buffer (10mM $Na_3C_6H_9O_9$, 0.05% Tween 20, pH=6.0) at 55°C overnight. Samples were then washed 3 times in

PBS and incubated in Blocking buffer (20% normal donkey serum, 0.3% Triton X-100, and 1 mM $CaCl_2$ in PBS) overnight at 37°C or 24-72h at 4°C. Tissues were then rinsed in PBS and stained for EdU using Click-iT™ EdU Cell Proliferation Kit for Imaging (Thermo Fisher Scientific) following the provided protocol. The samples were then washed 3 times for 15 min in PBS and incubated with primary antibodies for 24h at 37°C with agitation in Diluent buffer (1% normal donkey serum, 0.3% Triton X-100, and 1 mM $CaCl_2$ in PBS). Samples were washed 3 times for 30 min in PBS to remove excess of primary antibodies and were subsequently incubated with the secondary antibodies for 24h at 37°C with agitation. After that, samples were washed 3 times for 30 min in PBS and incubated in Ce3D tissue clearing solution (Biolegend cat# 427704) for 2h at RT and then mounted between 2 glass slides, edges were sealed with superglue, and clamped with binder clips prior until the glue set. Explants were imaged using Zeiss LSM 880 line-scanning confocal microscope (Carl Zeiss, Germany).

Western Blot analysis

Protein samples were collected from primary SAE cells that have been edited with a specified gRNA, fluorescently sorted, and expanded in SAGM or cultured in Pneumacult ALI for a specified number of days. Cells were lysed either using RIPA buffer (Sigma) for 10 min on ice or using a fractionation kit NE-PER (Thermo Fisher Scientific) following the manufacturer's protocol. Protein concentration was measured using Pierce BCA assay kit (Thermo Fisher Scientific). Samples were run on a SDS PAGE gel under reducing conditions and transferred onto Amersham Protran 0.45 nitrocellulose membrane (GE healthcare), which was subsequently probed using the specified antibodies and imaged using Ai600 imager (GE healthcare). Band intensity was analyzed using ImageJ and normalized to loading control and to the WT control.

Quantitative PCR analysis

RNA was isolated from cells using RNeasy kit (Qiagen) according to the provided protocol. cDNA was synthesized using high-capacity cDNA synthesis kit (Applied Biosystems) according to the manufacturer's instructions. qPCR reactions were set up using 10nM primers (see Table 2 for details), cDNA, water and POWER SYBR master mix (Applied Biosystems) and run on CFX connect Real-Time PCR detection system (Bio-Rad). The expression data was normalized using delta-delta-CT method.

Transwell migration assays

Migration assays were performed using primary mouse SAE or SMG cells grown in SAGM. Cells were seeded at a density of $3x10^5$ cell/per well (d=6.5 mm, pore size 8 μm. Corning, cat# 353097) in modified SAGM (without the TGFβ antagonist [1 μM A83-01] and with reduced BSA [0.5%]). The bottom transwell chamber contained modified SAGM with TGFβ1 (10 ng/ml, PeproTech), which served as a chemoattractant. Passage 1 Krt15-KO SAE and SMG isolated from Krt15 whole body KO mice were used in regular migration assays, while a ~1:1 mix of passage 4 Krt14-KO:WT cells was used in competitive migration assays. The exact ratios of Krt14-KO:WT were determined by staining chamber slides (Lab-Tek, Cat# 177445) which were processed in parallel to competitive migration assays. Cells were fixed in 4% PFA 12h after seeding and the underside of the membrane was subsequently stained for Krt5 and Krt14. Keratin staining area was quantified using ImageJ software.

Proximity ligation assay

Passage 2 primary Human SAE from 3 donors were seeded on permanox chamber slides (Lab-Tek, Cat# 177445). 12h later, cells were washed and fixed for 10 minutes in 4% PFA and blocked using the blocking buffer provided in the PLA kit (Millipore Sigma, Cat# DUO92202). The assay was carried out according to the provided protocol. Primary antibodies were incubated o/n at 4°C in individual chambers while all the subsequent steps of the protocol

were carried out after removing the chamber dividers. We used the following primary antibodies at the specified concentration: Krt14 (RB-9020-P1) 1:500; Sfn (1433s01) 1:50; Krt15 (HPA024554) 1:300. Experimental samples and negative controls (no primary antibody for each one or both target proteins) were run on the same slides. PLA signal was calculated by dividing the total PLA puncta (determined by intensity thresholding and object count module in Fiji) by the number of nuclei in maximum intensity projection images (24-26 z-planes spanning 24-26 μm of depth). At least 100 cells per donor were analyzed in each group.

Supplemental References

1. Swatek AM, Lynch TJ, Crooke AK, Anderson PJ, Tyler SR, Brooks L, et al. Depletion of Airway Submucosal Glands and TP63(+)KRT5(+) Basal Cells in Obliterative Bronchiolitis. *Am J Respir Crit Care Med.* 2018;197(8):1045-57.
2. Lynch TJ, Anderson PJ, Rotti PG, Tyler SR, Crooke AK, Choi SH, et al. Submucosal gland myoepithelial cells are reserve stem cells that can regenerate mouse tracheal epithelium. *Cell stem cell.* 2018;22(5):653-67. e5.

Table S1. Primary antibodies used

Antibody target	Company	Catalogue #	Concentration
Keratin 5	BioLegend	905901	1:500
Keratin 15	BioLegend	833901	1:200
Ac-Tubulin	Cell Signaling Technology	5335	1:500
Scgb1a1	Millipore Sigma	ABS1673	1:4000
p63	BioCare Medical	CM163A	1:300
dNp63	Stem Cell Technologies	60154	1:300
Keratin 14	Labvision	RB-9020-P1	1:500
Keratin 14	Thermo Fisher Scientific	MA5-11599	1:300
α-SMA	Abcam	ab7817	1:500
Yap	Thermo Fisher Scientific	PA1-46189	1:2000
Vinculin	Milipore Sigma	V 9131	1:2000
Lamin B1	Abcam	ab16048	1:1000
Beta-catenin	Abcam	ab6302	1:2000
14-3-3 sigma (Sfn)	Thermo Fisher Scientific	1433s01	1:50
Keratin 15	Milipore Sigma	HPA024554-100UL	1:300

Table S2 Primers and gRNAs used

LoxP gRNA	GTATGCTATACGAAGTTATT
Tomato gRNA	CAAAGAGTTCATGCGCTTCA
Krt14 gRNA	ACCCACCTGGCCTCTCATGG
Krt15 gRNA	CAAACGTCTTCCACTTTTGG
Krt15 qPCR primers	Fwd: TGGCAGAGATGAGGGAGCAGTA<br>Rev: GTGTTAGACGCCACCTCCTTGT
Krt14 qPCR primers	Fwd: GAAGAACCGCAAGGATGCTGAG<br>Rev: TGCAGCTCGATCTCCAGGTTCT
CYR61 qPCR primers	Fwd: GTGAAGTGCGTCCTTGTGGACA<br>Rev: CTTGACACTGGAGCATCCTGCA
Axin2 qPCR primers	Fwd: GAGCAGCCTTGACCAGTCTC<br>Rev: GAGGAGCCGTTCTGAATCTG
P63 qPCR primers	Fwd GTATCGGACAGCGCAAAGAACG<br>Rev CTGGTAGGTACAGCAGCTCATC
dNp63 qPCR primers	Fwd: GAGCAGCCTTGACCAGTCTC<br>Rev: GAGGAGCCGTTCTGAATCTG
P21 qPCR primers	Fwd: TCGCTGTCTTGCACTCTGGTG<br>Rev: CCAATCTGCGCTTGGAGTGATAG
ActinB qPCR primers	Fwd: CATTGCTGACAGGATGCAGAAGG<br>Rev: TGCTGGAAGGTGGACAGTGAGG

Supplemental figures and legends:

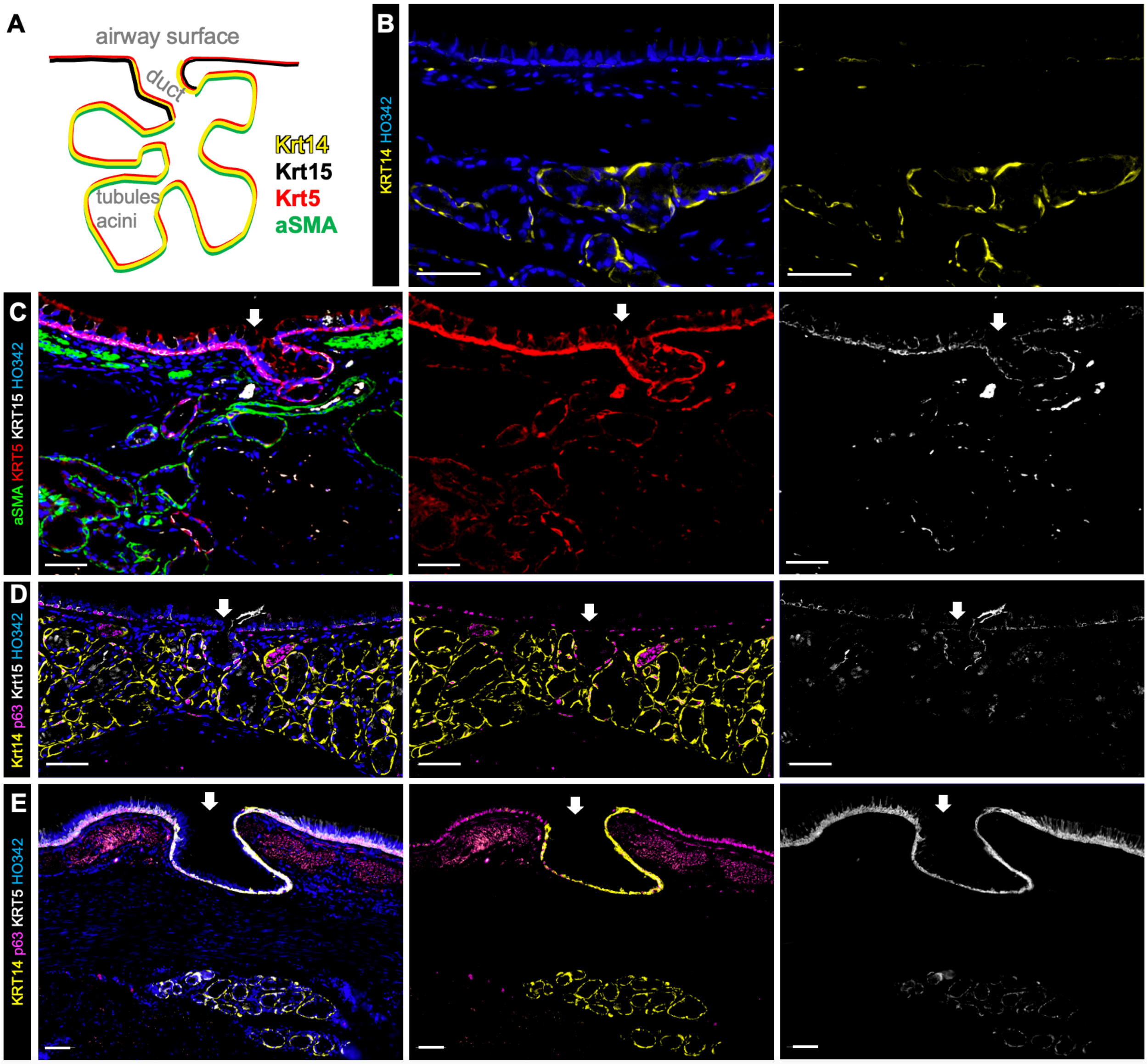

Figure S1. **Basal keratin and αSMA expression in the large airways of ferrets, mice, and humans.**

(**A**) Schematic representation of Krt14, Krt15, Krt5 and αSMA expression pattern in large airways. (**B**) Immunofluorescent staining of wild-type adult ferret pulmonary bronchus with KRT14. (**C**) Wild-type adult ferret pulmonary bronchus stained with αSMA, KRT5 and KRT15. (**D**) Wild-type 10-week-old mouse trachea stained with Krt14, p63 and Krt15. (**E**) Healthy adult human pulmonary bronchus stained with KRT14, p63 and KRT5. Arrows mark glandular ducts. Images are representative of N≥3 animals or human patients. Scale bars in **B-D** are 50 μm, scale bars in **E** are 100μm.

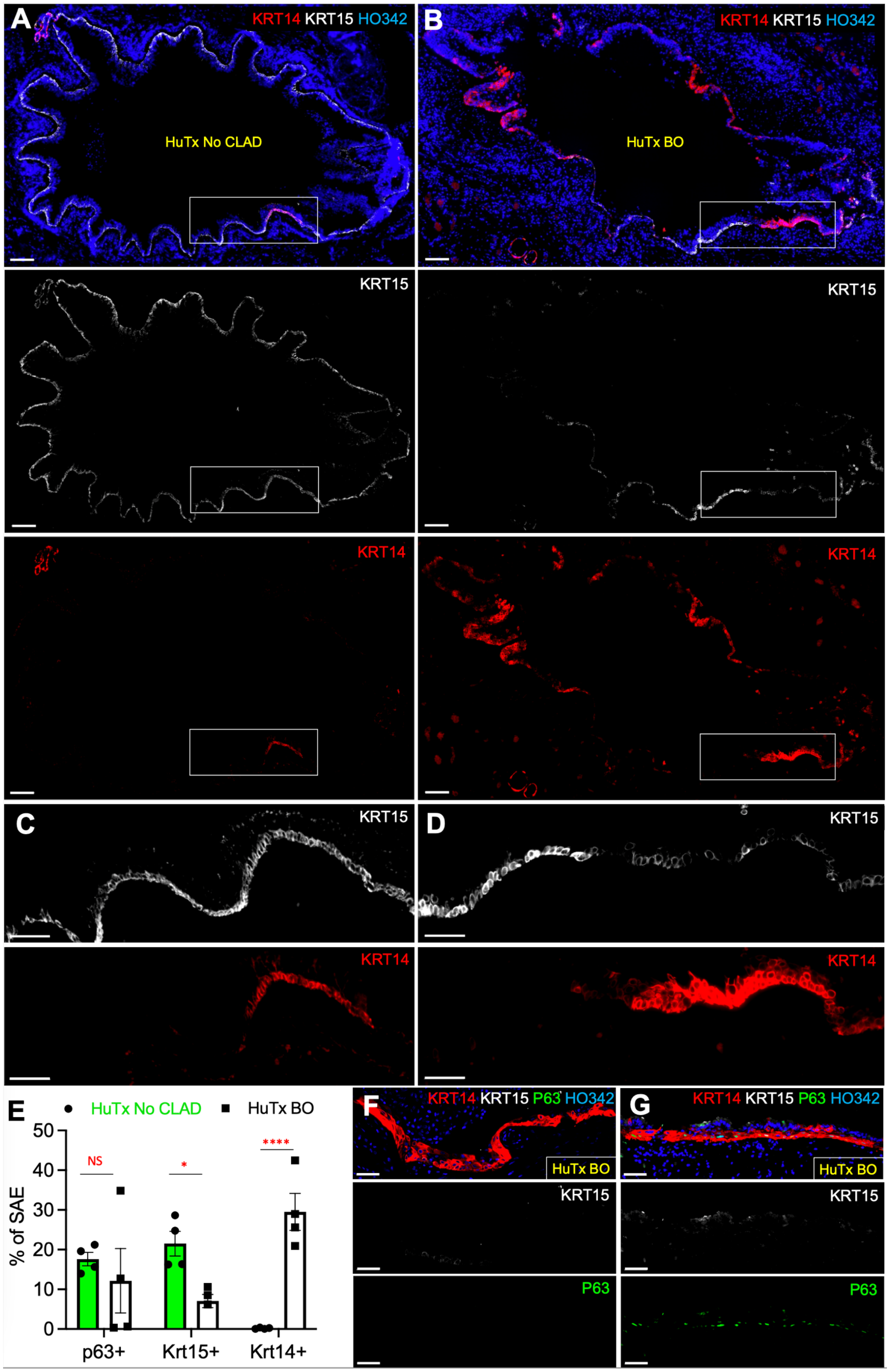

Figure S2. **KRT14 displaces KRT15 in Human BO airways.**
(**A**) Immunofluorescent staining of an intermediate airway from a human lung transplant recipient who did not develop chronic lung allograft dysfunction (No CLAD). (**B**) Staining of an intermediate airway from a human lung transplant recipient who developed bronchiolitis

obliterans (BO). (**C, D**) Magnified regions from panels **A** and **B**. (**E**) Quantification of p63, Krt14 and Krt15 expression in large and medium airways. Graphs show mean +/- SEM, N=4 human patients. (**F-G**) Two additional independent examples of human BO airways. Significance was determined by a two-way ANOVA and Benjamini Hochberg FDR (0.05) method. * – *P*<0.05, **** – *P*<0.0001. Scale bars are 100 µm in **A** and **B**, and 50 µm in panels **C,D,F,G**.

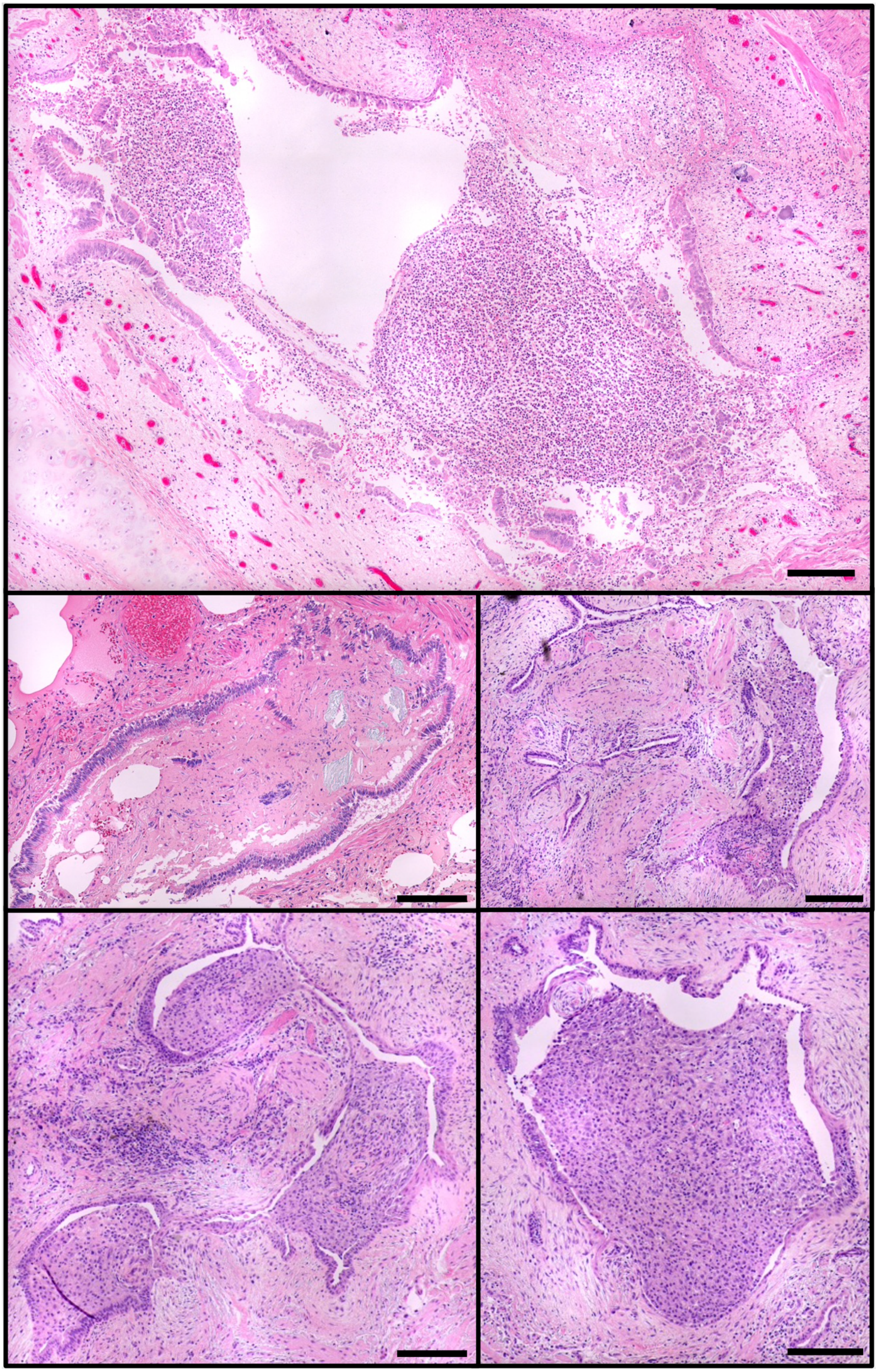

Figure S3. **Histopathology of Human BO lesions.**
H&E staining of small airways of human patients who developed BO after lung transplantation. N=4 patients. Scale bars are 200 μm.

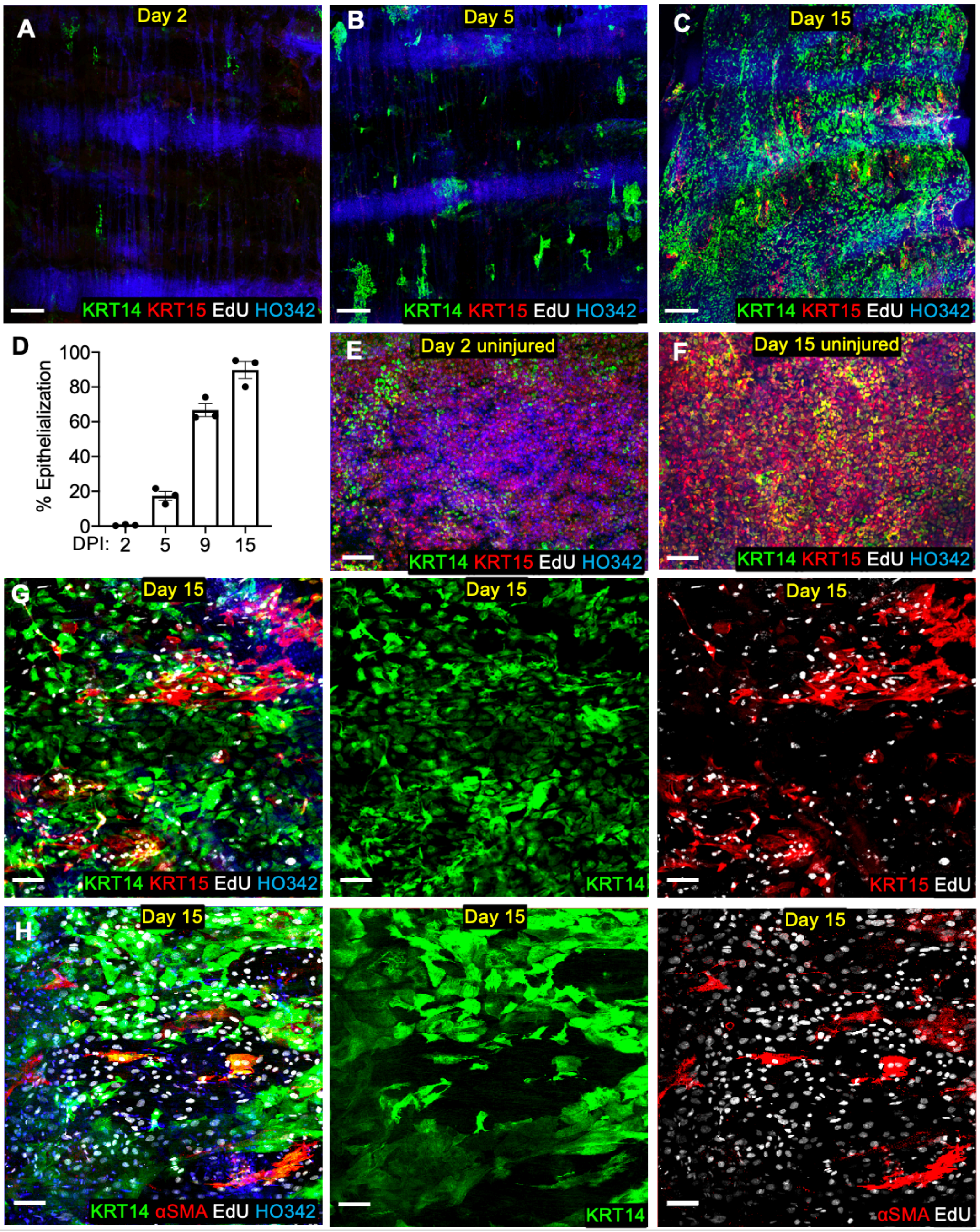

Figure S4. **Re-epithelialization of the tracheal surface after a brush injury ex vivo.** (**A-C**) Representative examples of epithelialization of ferret tracheal explants at 2, 5 and 15 days post mechanical brushing injury. The entire luminal surface was brushed off, explants were pulsed with EdU for 24h on day 3, except in panel H the explants were cultured in EdU continuously. (**D**) Quantification of epithelialization of the tracheal surface over time. (**E-F**) Representative images of the SAE from uninjured explant on day 2 and day 15 in ex vivo culture. (**G-H**) Representative images of explants recovering from a total brush injury on day 15. Graphs show mean +/- SEM, N=3 explants from independent ferrets. Scale bars in **A-C** are 500 μm, while scale bars in **E-H** are 100 μm.

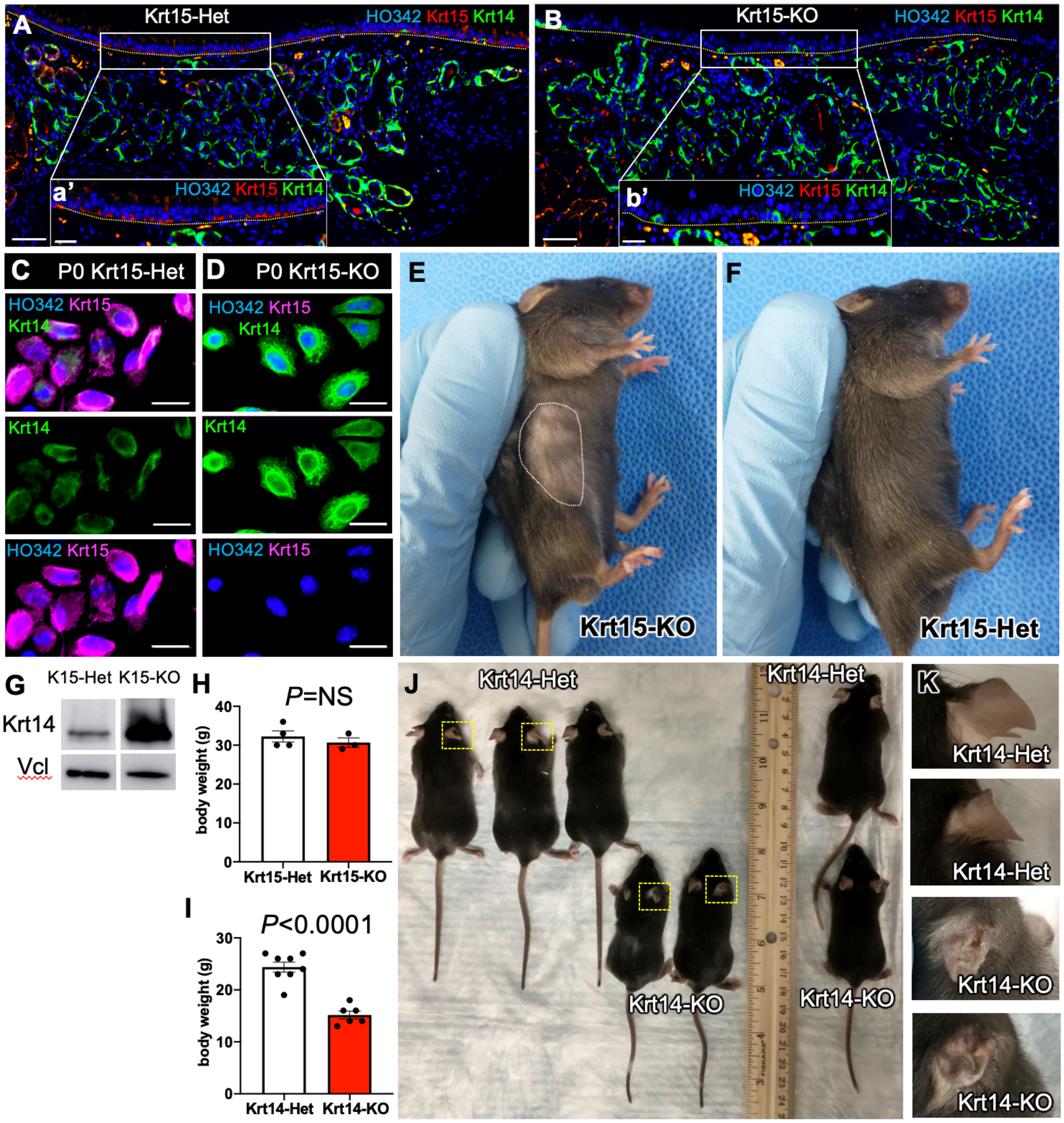

Figure S5. **Initial phenotyping of Krt15-KO and Krt14-KO mice**
(**A-B**) Immunofluorescent staining of the SMG region of the proximal trachea of Krt15-Het (**A**) and Krt15-KO (**B**) 8-week-old mice. (**a'-b'**) Indicated regions of the SAE are magnified. Yellow regions below the basement membrane (dotted line) are auto-fluorescent red blood cells. (**C-D**) Immunofluorescent staining passage 0 primary SAE from Krt15-Het (**C**) and Krt15-KO (**D**) mice. (**E-F**) Whole body Krt15-KO mice are viable and fertile. Adult homozygous Krt15-KO mice (past 8 weeks) display partial hair loss (**E**), while Krt15-Het mice appear phenotypically normal (**F**). (**G**) Western blot analysis of primary airway basal cells isolated from 3 independent Krt15-KO mice. (**H**) 10-week-old Krt15-KO and Krt15-Het mice have similar body weight. (**I-K**) 8-week-old Krt14-KO mice have a significant weight reduction compared to Krt14-Het littermates, few Krt14-KO mice survive past weaning, but have frequent ear defects (**K**). Graphs show mean +/- SEM, N≥3 independent mice. Images are representative of N≥3 animals. Significance was determined by 2-tailed T-test. Scale bars are 20µm, except in **A** and **B** are 50 µm.

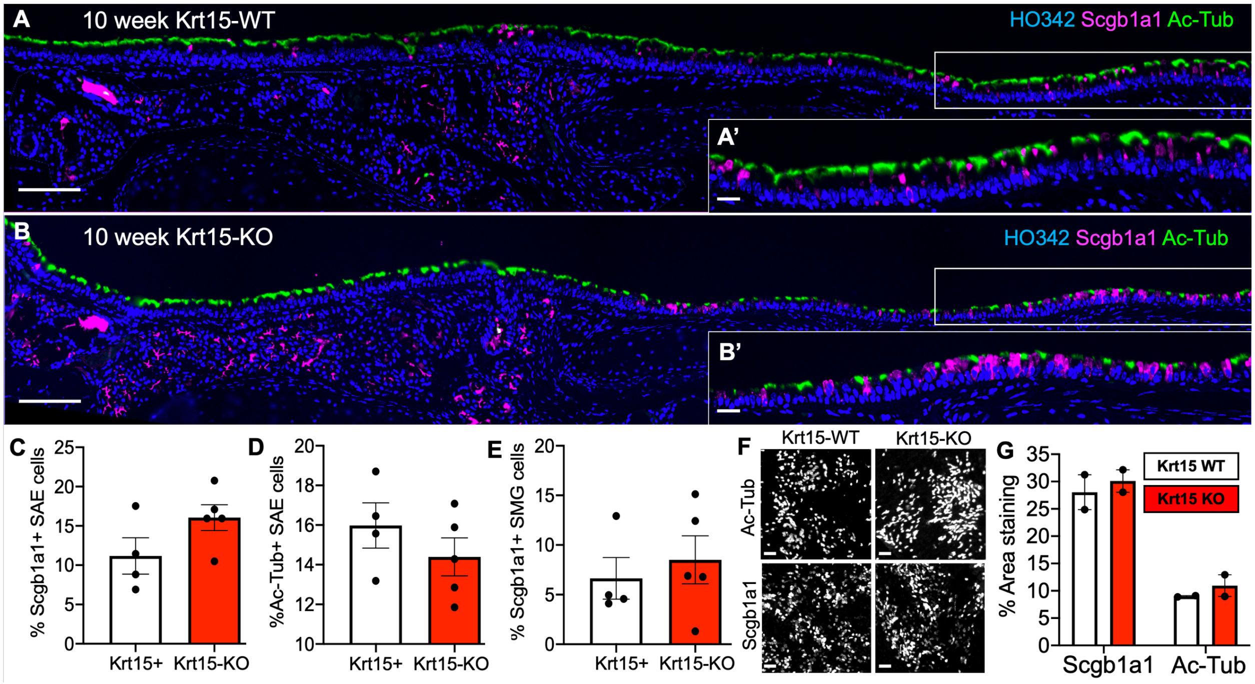

Figure S6. **Krt15-KO does not affect club and ciliated cell differentiation *in vivo* and *in vitro*.**

(**A-B**) Representative confocal micrographs of 10-week-old Krt15-WT (**A**) and Krt15-KO (**B**) mouse tracheas. (**A-B'**) Close up micrographs of the indicated regions in A and B. (**C-E**) Quantification of secretory (**C,E**) and ciliated (**D**) cell abundance in the SAE (**C-D**) and the SMG (**E**). Krt15+ controls include 2 Krt15-WT and 2 Krt15-Het animals. (**F**) Representative micrographs of passage 3 Krt15-WT and Krt15 Crispr KO cells differentiated for 21 days on the ALI and stained for Scgb1a1 and Ac-Tub. (**G**) Quantification of staining in F. Graphs in **C-D** show mean +/- SEM, N≥4 animals. Graph in **G** shows mean +/- SEM, N=2 differentiated transwells grown from a cell pool of 3 animals. Significance was determined using 2-tailed T-test in C-E or by one-way ANOVA, Tukey multiple comparison test in G. Scale bars are 100 µm in all panels, except **A'** and **B'** scale bars are 20 µm.

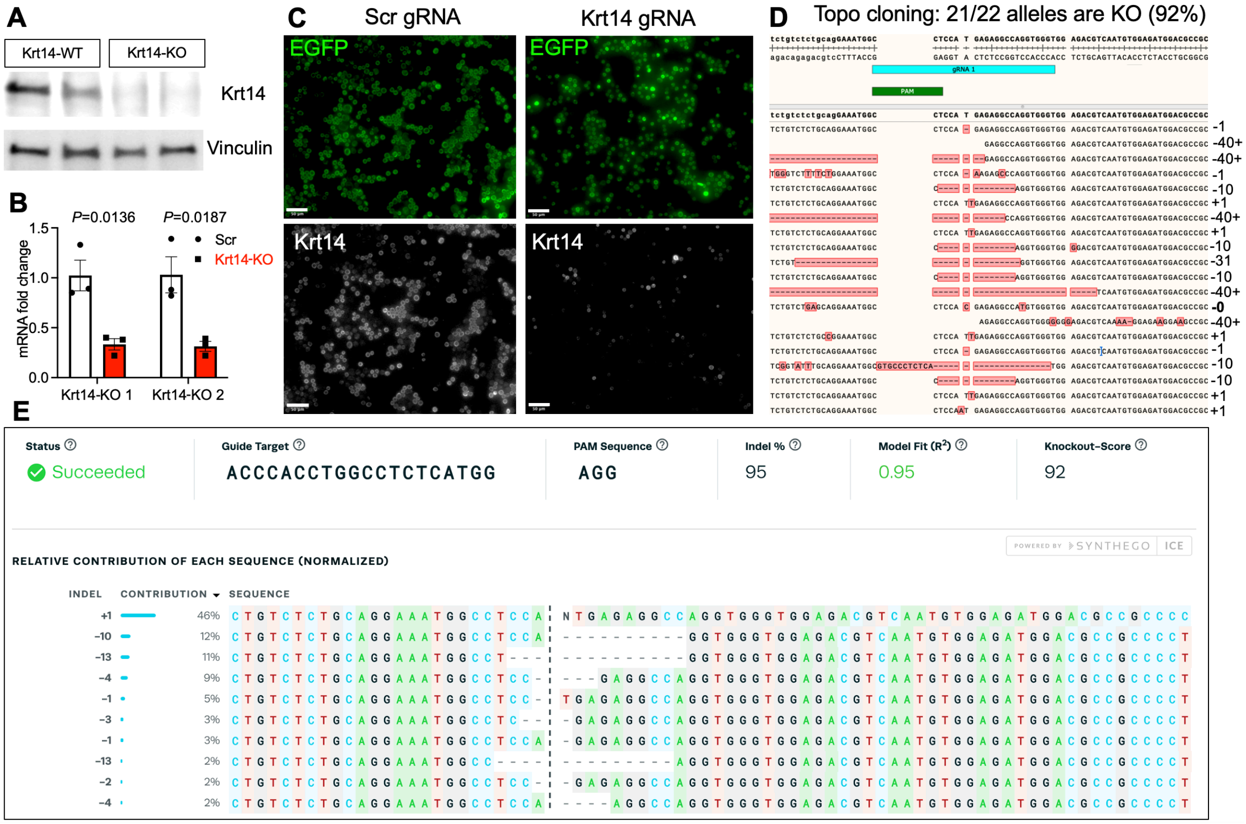

Figure S7. **Validation of Krt14 CRISPR KO in primary airway basal cells**
Passage 5 cells from Rosa-TG:H11-Cas9 mice were co-transfected with a mixture of Krt14 and LoxP gRNAs and FACS sorted for EGFP+ population 7 days later. (**A-B**) Cells from 2 independent representative transfections were used for western blotting (**A**) and for qPCR analysis (**B**). (**C-E**) Cells from 1 representative transfection were used for immunofluorescent staining after cytospinning (**C**) and for Topo-cloning analysis of Krt14 locus after DNA isolation and PCR (**D**). (**E**) Synthego CRISPR ICE analysis of the sequenced Krt14 locus isolated from the population of Krt14 gRNA- treated, FACS sorted cells. Graphs show mean +/- SEM, representative of N≥4 experiments. Significance was determined using 2-tailed T-test. Scale bars are 50 μm.

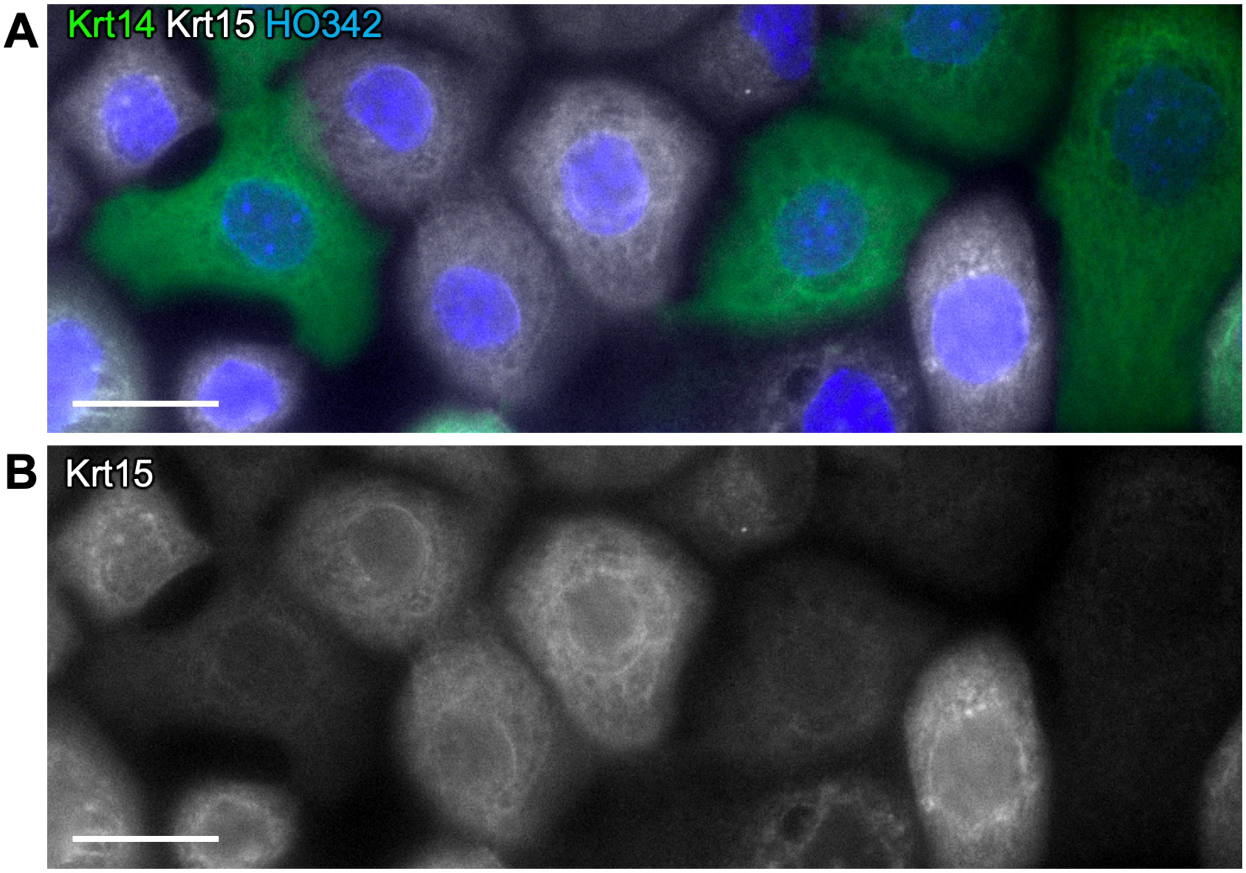

Figure S8. **Krt15 gets upregulated in advanced passage Krt14-KO cells.**
(**A-B**) Immunofluorescent micrographs of passage 8 primary SAE from ROSA-TG:H11-Cas9 mice that had been transfected with a mixture of Krt14 and Tomato gRNAs, FACS sorted for Tomato- and stained for Krt14 and Krt15. Composite (**A**) and Krt15 channel alone (**B**). Images are representative of N≥3 experiments. Scale bars are 25 µm.

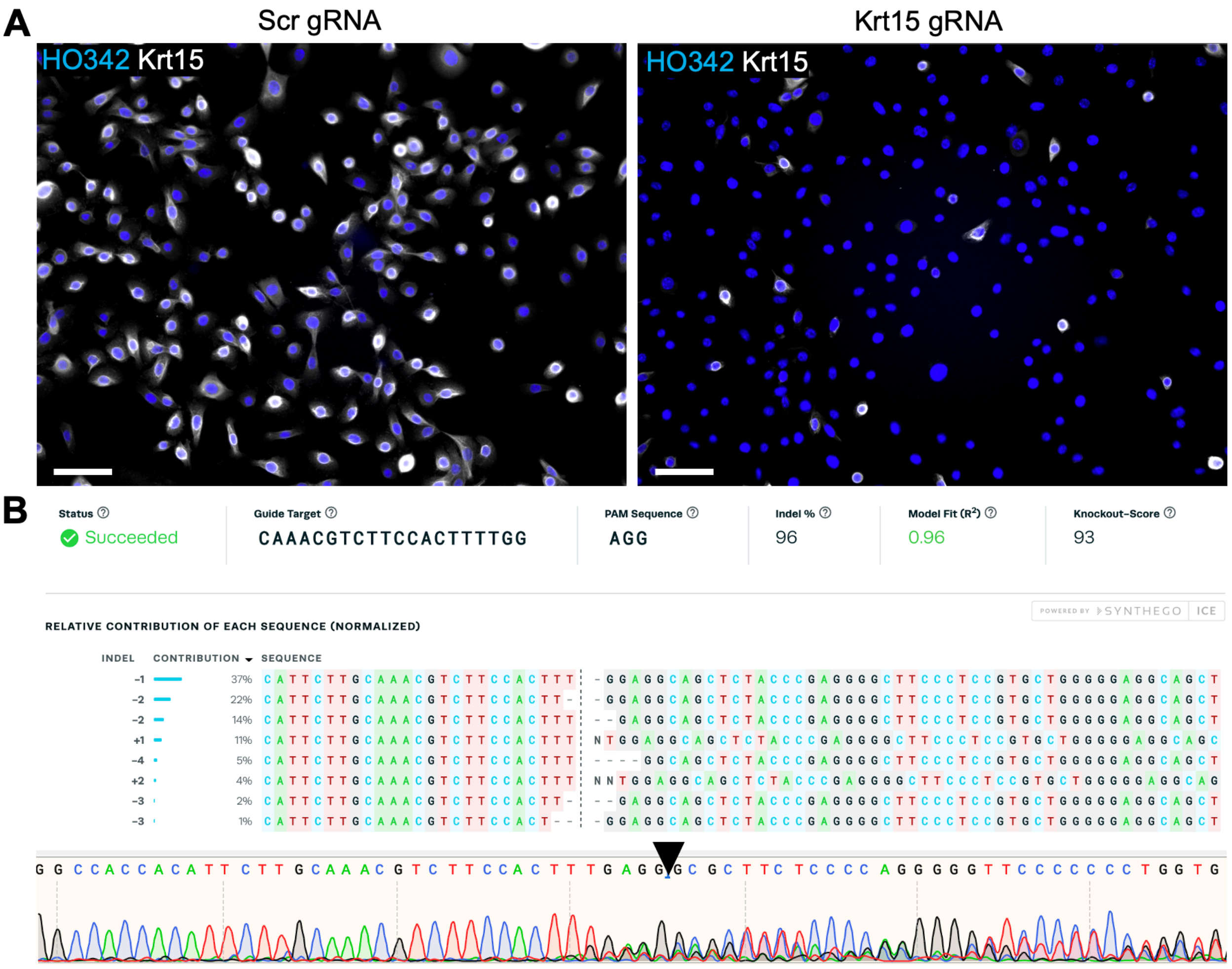

Figure S9. **Verification of Krt15 CRISPR/Cas9 knockout efficiency**
(**A**) Immunofluorescent staining of primary airway BCs that were transfected with Krt15 or Scrambled gRNA and LoxP gRNA on passage 2, FACS sorted for EGFP+ cells 2 days later and for Krt15. (**B**) Synthego CRISPR ICE analysis shows the knockout score of 93% for the sorted EGFP+ Krt15-KO cell population. Arrowhead indicates the end of PAM sequence (AGG). Images are representative of N≥3 experiments. Scale bars are 50 µm.

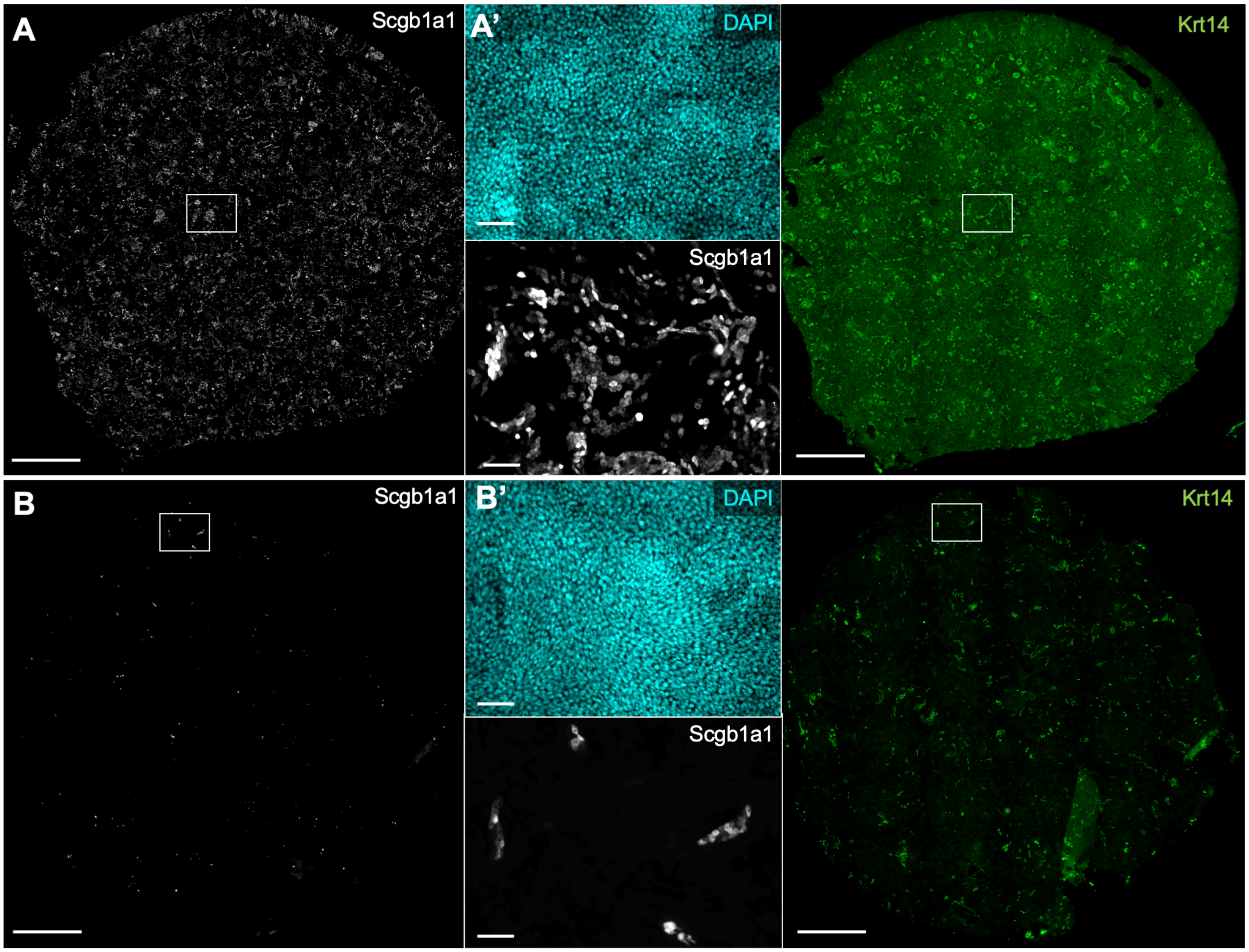

Figure S10. **Krt14-KO ALI cultures have impaired club cell differentiation.**
(**A**) Immunofluorescent staining of day 21 ALI cultures from Tomato/Scrambled gRNA-transfected FACS sorted (Tomato-) basal cells. (**B**) Immunofluorescent staining of day 21 ALI cultures from Tomato/Krt14 gRNA-transfected FACS sorted (Tomato-) basal cells. (**A'-B'**) Indicated regions are magnified in **A'** and **B'**. Images are representative of N≥3 experiments. Scale bars in **A** and **B** are 1000µm, Scale bars in **A'** and **B'** are 100 µm.

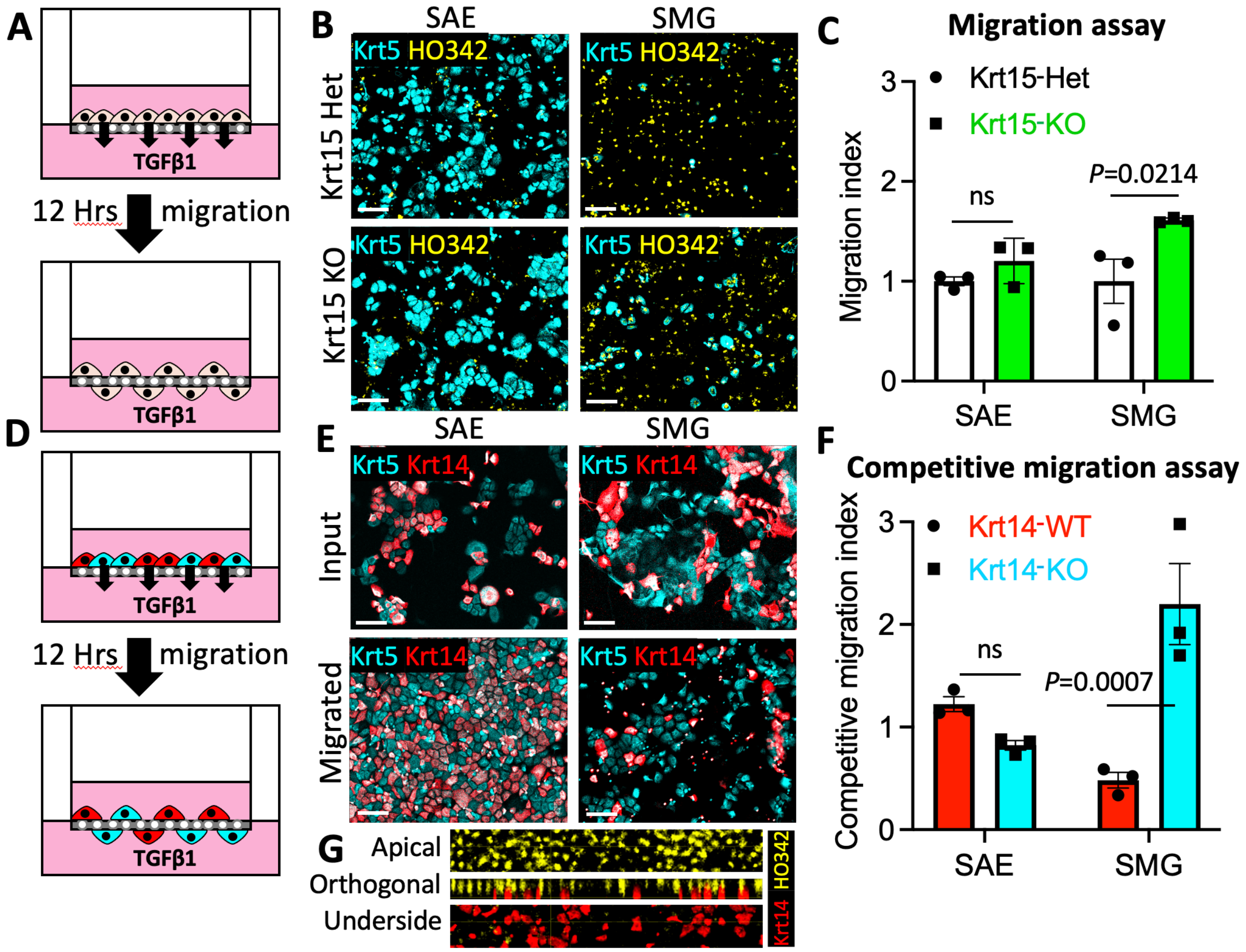

Figure S11. **Loss of Krt14 and Krt15 improves glandular progenitor cell migration *in vitro*.** (**A**) Experimental design of migration assay: passage 1 Krt15-KO or Krt15-Het cells were seeded to confluency (3*10^5 cells/well) into 804G-coated 6.5mm transwells (pore size 8µm). Cells were allowed to migrate for 12h through the pores to the underside of the transwell towards the chemoattractant TGFβ1 (10 ng/ml) after which the cells on the underside were fixed and stained for Krt5. (**B**) Confocal micrographs of the migrated cells. (**C**) Quantification of migrated cell abundance on the underside of the transwell normalized to WT controls. (**D**) Experimental design of competitive migration assay: passage 4 Krt14-KO and Krt14-WT cells were mixed at ~1:1 ratio and seeded to confluency (3*10^5 cells/well) into 804G-coated 6.5mm transwells (pore size 8µm). Cells were allowed to migrate for 12h through the pores to the underside of the transwell towards the chemoattractant TGFβ1 (10 ng/ml). In parallel, the same mix of cells was seeded on chamber slides for 12h after which the chamber slides and the underside of the transwells were both fixed and stained for Krt14 and Krt5. (**E**) Confocal micrographs of the Krt14-KO:WT 1:1 mix of cells when seeded on chamber slides (input) and after migration to the underside of the transwell (migrated). (**F**) Quantified ratio of Krt14-KO:WT in migrated cells/ input cells (competitive migration index). (**G**) Confocal z-stacks and orthogonal view projection showing that only the underside of the transwells was stained with Krt14. Graphs show mean +/- SEM, N=3 donor cell pools from 1-3 mice each. Statistical significance was determined by two-way-ANOVA, Tukey's multiple comparison test. Scale bars are 100 µm.
